# Two New Succinimide Derivatives Cladosporitins A and B from the Mangrove-derived Fungus *Cladosporium* sp. HNWSW-1

**DOI:** 10.3390/md17010004

**Published:** 2018-12-20

**Authors:** Pei Wang, Yan Cui, Caihong Cai, Huiqin Chen, Yu Dai, Pengwei Chen, Fandong Kong, Jingzhe Yuan, Xinming Song, Wenli Mei, Haofu Dai

**Affiliations:** 1 Key Laboratory of Biology and Genetic Resources of Tropical Crops, Ministry of Agriculture, Institute of Tropical Bioscience and Biotechnology, Chinese Academy of Tropical Agricultural Sciences, Haikou 571101, China; wangpei@itbb.org.cn (P.W.); cuiyan502@163.com (Y.C.); caicaihong@itbb.org.cn (C.C.); chenhuiqin@itbb.org.cn (H.C.); daiyu15@mails.ucas.ac.cn (Y.D.); chenpengwei@itbb.org.cn (P.C. kongfandong@itbb.org.cn (F.K.); yuanjingzhepc@126.com (J.Y.); 2 Hainan Key Laboratory for Research and Development of Natural Product from Li Folk Medicine, Haikou 571101, China; 3 Key Laboratory of Tropical Medicinal Plant Chemistry of Ministry of Education and Hainan Normal University, Haikou 571101, China; sxm8646@163.com

**Keywords:** mangrove-derived fungus, *Cladosporium* sp., succinimide-containing derivatives, cytotoxicity, α-glycosidase inhibitor

## Abstract

Two new succinimide-containing derivatives, cladosporitins A (**1**) and B (**2**), were isolated from the fermentation cultures of the mangrove-derived fungus *Cladosporium* sp. HNWSW-1, along with a new pyrone, clapone (**3**), as well as the previously reported talaroconvolutin A (**4**) and anthraquinone (**5**). The structures of the isolated compounds were elucidated by 1D, 2D NMR, and HRMS spectral analysis. Compound **2** showed cytotoxicity against BEL-7042, K562 and SGC-7901 cell lines with IC_50_ values of 29.4 ± 0.35 μM, 25.6 ± 0.47 μM, and 41.7 ± 0.71 μM, respectively, whereas compound **4** exhibited cytotoxicity against Hela and BEL-7042 cell lines with IC_50_ values of 14.9 ± 0.21 μM and 26.7 ± 1.1 μM, respectively. In addition, compounds **4** and **5** displayed inhibitory activity against α-glycosidase, with IC_50_ values of 78.2 ± 2.1 μM and 49.3 ± 10.6 μM, respectively.

## 1. Introduction

Fungi are an important resource of structurally and biologically diverse substances for drug and pesticide discovery [1,2,3,4,5,6,7]. A set of rare natural compounds from fungi, such as talaroconvolutins A-D, ZG-1494R, oteromycin, and codinaeopsin, which contain a modified tetramic acid unit linked to phenol, benzene, or indole and decalin fragments, exhibit activities against fungi, platelet-activating factors, acetyltransferase, endothelins receptors, or *Plasmodium falciparum* [8,9,10,11]. During our ongoing search for new bioactive metabolites from marine fungi, *Cladosporium* sp. HNWSW-1 was isolated from the healthy tree root of *Ceriops tagal* collected from the Dong Zhai Gang Mangrove Reserve in Hainan. The secondary metabolites of the genus *Cladosporium* have been mainly reported as polyketides derivatives, such as fatty acids [12], macrolides [13,14,15], pyrones [16,17,18], binaphthyl derivatives [19,20], α-pyridone [21] and tetramic acid derivatives [22,23]. Subsequent chemical investigations on the EtOAc extract of its fermentation cultures led to the isolation of cladosporitins A (**1**) and B (**2**), which contain the succinimide unit linked to phenol and decalin fragments. In addition, a new pyrone, clapone (**3**), was also isolated along with the previously reported talaroconvolutin A (**4**) [8] and 1,3,6-trihydroxy-7-(1-hydroxyethyl) anthracene-9,10-dione (**5**) [24]. Herein, we describe the isolation, structural determination, and biological activities of compounds **1**–**5**.

## 2. Results

### 2.1. Structural Elucidation

**Figure 1:**
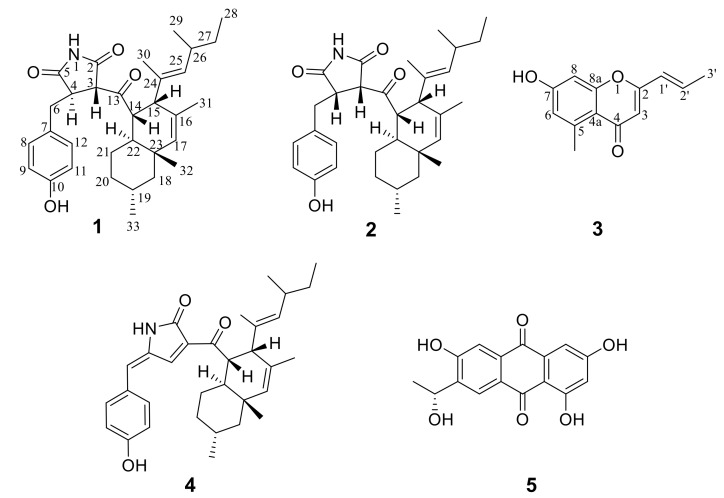
Chemical structures of compounds **1**–**5** from *Cladosporium* sp. HNWSW-1.

Compound **1** was isolated as yellow oil. Its molecular formula was determined as C_32_H_43_NO_4_ by HRESIMS *m*/*z* 528.3103 [M + Na]^+^ (calcd. for C_32_H_43_NO_4_Na 528.3084) (Figure S18 in Supplementary Materials). The ^1^H, DEPTQ and HSQC NMR spectra (Figures S1, S2, and S6–S8 in Supplementary Materials) of compound **1** showed signals of one ketone carbonyl at *δ*_C_ 203.9, two amide carbonyls at *δ*_C_ 172.7 and *δ*_C_ 179.2, a *para*-substituted benzene ring (*δ*_C_ 131.5/ *δ*_H_ 7.10, *δ*_C_ 116.3/*δ*_H_ 6.77, *δ*_C_ 157.5 and *δ*_C_ 128.1), seven *sp^3^* methines (*δ*_C_ 61.7, *δ*_C_ 52.9, *δ*_C_ 51.8, *δ*_C_ 45.0, *δ*_C_ 40.8, *δ*_C_ 34.8, and *δ*_C_ 28.1), two *sp^2^* methines (*δ*_C_ 137.1 and *δ*_C_ 136.7), five methylenes (*δ*_C_ 49.1, *δ*_C_ 36.5, *δ*_C_ 35.8, *δ*_C_ 31.1, and *δ*_C_ 24.1), and six methyls (*δ*_C_ 23.1, *δ*_C_ 22.5, *δ*_C_ 21.1, *δ*_C_ 20.5, *δ*_C_ 15.2, and *δ*_C_ 12.4) (Table 1), except in the above data there were also three quaternary carbons (*δ*_C_ 130.7, *δ*_C_ 135.7, and *δ*_C_ 36.2). The comparison of ^1^H and ^13^C NMR data (Table 1) of the previously reported talaroconvolutin A (**4**) [8] revealed many similarities. Compound **1** had the same decalin moiety with the substituent that is the 4-methylhex-2-en-2-yl substituent linked to C-15 (*δ*_C_ 51.8) as compound **4**. This is evidenced by the sequential COSY correlations (Figures S3–S5 in Supplementary Materials) from H-15 (*δ*_H_ 3.10) through H_3_-33 (*δ*_H_ 0.82), and H_2_-18 (*δ*_H_ 1.48/0.87), from H_3_-28 (*δ*_H_ 0.86) through H-25 (*δ*_H_ 5.05) and H_3_-29 (*δ*_H_ 0.92), as well as by HMBC correlations (Figures S9–S14 in Supplementary Materials) from H-14 (*δ*_H_ 3.62) to C-23 (*δ*_C_ 36.2), and C-24 (*δ*_C_ 135.7), from H-15 to C-17 (*δ*_C_ 137.1), C-22 (*δ*_C_ 40.8), C-24, C-30 (*δ*_C_ 15.2), and C-31 (*δ*_C_ 22.5), from H-17 (*δ*_H_ 5.38) to C-15, C-18 (*δ*_C_ 49.1), C-31, and C-32 (*δ*_C_ 20.5), from H_2_-18 to C-20 (*δ*_C_ 36.5), C-22, and C-33 (*δ*_C_ 23.1), from H-22 (*δ*_H_ 1.78) to C-20, C-21 (*δ*_C_ 24.1), and C-23, from H_3_-31 (*δ*_H_ 1.54) to C-15, C-16 (*δ*_C_ 130.7), and C-17, from H_3_-33 to C-18 and C-20, from H_3_-32 (*δ*_H_ 0.91) to C-17, C-18, C-22, and C-23, from H-25 to C-15, C-26 (*δ*_C_ 34.8), C-27 (*δ*_C_ 31.1), C-29 (*δ*_C_ 21.1), and C-30 (*δ*_C_ 15.2), from H-26 (*δ*_H_ 2.25) to C-27, C-28 (*δ*_C_ 12.4), and C-29, as well as from H_3_-29 to C-25 (*δ*_C_ 136.7), C-26, and C-27. The differences between compounds **1** and **4** are that the modified tetramic acid unit, 1,5-dihydro-2*H*-pyrrol-2-one unit, and C-6/C -7 double bond in compound **4** were replaced by the succinimide unit and CH_2_-6/CH-7 unit in compound **1**, respectively, as deduced by the sequential COSY correlations (Figures S3–S5 in Supplementary Materials) from H-3 (*δ*_H_ 4.06) to H_2_-6 (*δ*_H_ 3.11/2.87) through H-4 (*δ*_H_ 3.40) and the HMBC correlations (Figures S9–S14 in Supplementary Materials) from H-3 to C-2 (*δ*_C_ 172.7)/C-4 (*δ*_C_ 45.0)/C-5 (*δ*_C_ 179.2)/C-6 (*δ*_C_ 35.8), from H-4 to C-2/C-3 (*δ*_C_ 61.7)/C-5/C-6/C-7 (*δ*_C_ 128.1), and from H_2_-6 to C-3/C-4/C-5/C-7/C-8 (*δ*_C_ 131.5). In addition, the COSY and HMBC correlations suggested that the *para*-disubstituted benzene ring was linked to C-4 in compound **1** via C-6. Finally, the key HMBC correlations from H-3 and H-4 to C-13 (*δ*_C_ 203.9) and from H-14 to C-13 confirmed that the decalin and the succinimide fragments were linked through a ketone carbonyl (C-13). According to the molecular formula and the chemical shift of C-10 (*δ*_C_ 157.5) in compound **1**, a hydroxyl group was present on C-10. ROESY correlations (Figures S15–S17 in Supplementary Materials) from H-14 and H-19 to H_3_-32 suggested the relative configurations of C-14, C-19, and C-23 as shown in Figure 1. The large coupling constant (*J* = 12.0 Hz) of H-14/H-22 indicated their *trans*-diaxial orientation, while a coupling constant of 7.0 Hz between H-14 and H-15 placed these two protons in an axial-equatorial orientation [9,11]. Moreover, the ROESY correlation from H-3 to H_2_-6 (*δ*_H_ 2.87) hinted that H-3 and H-4 were on opposite sides of the succinimide ring. ROESY correlations (Figures S15–S17 in Supplementary Materials) from H_3_-30 to H-26 and from H-25 to H-15 proved the *E*-configuration of Δ^24^ double bond. Thus, the structure of compound **1** was established as shown in Figure 1 and was named cladosporitin A. However, the relative relationship between the chiral carbons in the succinimide fragment and those in the decalin fragment cannot be determined due to the free rotation of the C-3/C-13/C-14 linkage. 

**Figure 2:**
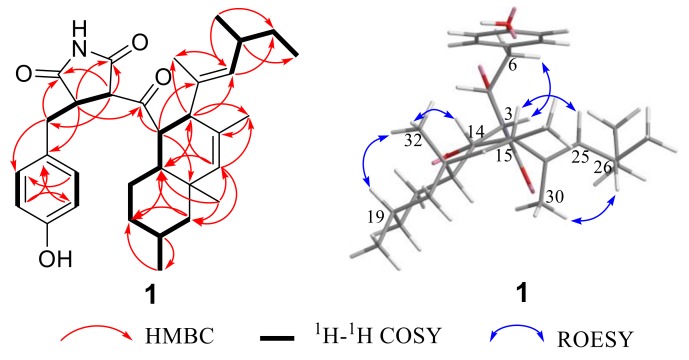
The key 2D NMR correlations for compound **1.**

Compound **2** was also isolated as a yellow oil with the same molecular formula as compound **1**, as determined by the HRESIMS peak at *m*/*z* 506.3279 [M + H]^+^ (calcd. for C_32_H_4__4_NO_4_ 506.3265) (Figure S39 in Supplementary Materials). A detailed comparison of NMR data of compound **2** (Figures S19–S36 in the Supplementary Materials and Table 1) with those of compound **1** indicated that compound **2** had the same planar structure as compound **1**. ROESY correlations (Figures S37 and S38 in Supplementary Materials and Figure 2) from H-14 and H-19 to H_3_-32 along with the coupling constant of H-14/H-22 (*J* = 12.1 Hz) and H-14/H-15 (*J* = 6.9 Hz) suggested that the relative configurations of C-14, C-15, C-19, C-22, and C-23 in the decalin fragment of compound **2** were the same as those of compound **1**. However, the relatively large coupling constant between H-3 and H-4 of compound **2** (*J* = 4.1 Hz) compared to compound **1** (*J* = 2.4 Hz) combined with the absence of the ROESY correlation of H-3/H_2_-6 (*δ*_H_ 3.10/2.79) in compound **2** suggested the *cis* orientation of H-3 and H-4, which is different than that of compound **1**. Thus, compound **2** was elucidated as shown in Figure 1 and named cladosporitin B. 

**Figure 3:**
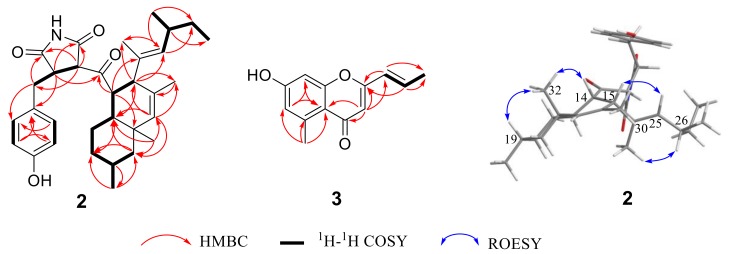
The key 2D NMR correlations for compounds **2** and **3**.

Compound **3**, a yellow oil, exhibited a prominent sodium adduct ion peak at *m/z* 239.0676 [M + Na]^+^ in the HRESIMS spectrum (Figure S50 in Supplementary Materials), suggesting a molecular formula of C_13_H_12_O_3_. Analysis of ^1^H NMR, ^13^C NMR, and HSQC spectra (Figures S40–S42 and S44–46 in Supplementary Materials) displayed five aromatic or olefinic methines (*δ*_C_ 137.3/*δ*_H_ 6.86, *δ*_C_ 124.8/*δ*_H_ 6.26, *δ*_C_ 118.3/*δ*_H_ 6.64, *δ*_C_ 109.9/*δ*_H_ 6.01, and *δ*_C_ 101.8/*δ*_H_ 6.70), two methyl siglets (*δ*_C_ 23.1/*δ*_H_ 2.73 and *δ*_C_ 18.6/*δ*_H_ 1.99), three sp^2^ oxyquaternary carbons (*δ*_C_ 164.0, *δ*_C_ 162.0, and *δ*_C_ 161.0), two sp^2^ quaternary carbons (*δ*_C_ 143.6 and *δ*_C_ 115.8), and one carbonyl (*δ*_C_ 182.3) (Table 2). The ^1^H NMR and ^13^C NMR data of compound **3** (Table 2) were very similar to those of 7-hydroxy-5-methyl-2- (2-oxobutyl)-4*H*-chromen-4-one [25]. The only major difference between them was the substituent linked to the 7-hydroxy-5-methyl-4*H*-chromen-4-one nucleus on C-2. The COSY correlations (Figure S43 in Supplementary Materials) of H-1′ (*δ*_H_ 6.26)/H-2′ (*δ*_H_ 6.86)/ H-3′ (*δ*_H_ 1.99), along with the HMBC correlations (Figures S47–S49 in Supplementary Materials and Figure 3) from H-1′ to C-2 (*δ*_C_ 162.0) and C-3′ (*δ*_C_ 18.6), from H-2′ to C-2 and C-3′, as well as from H-3 to C-1′ (*δ*_C_ 124.8) proved that a propylene fragment was located at C-2 in compound **3**. In addition, the large coupling constant (*J* = 15.6) between H-1′ and H-2′ deduced the *E*-configuration of Δ^1′^ double bond. Hence, compound **3** was identified and named clapone.

### 2.2. The Bioactivities of Compounds **1**–**5** from Cladosporium sp. HNWSW-1

Compounds **1**–**5** were tested for their cytotoxicity against Hela, BEL-7042, K562 and SGC-7901 cell lines (Table 3). Compound **2** showed cytotoxicity against the BEL-7042, K562 and SGC-7901 cell lines with IC_50_ values of 29.4 ± 0.35 μM, 25.6 ± 0.47 μM and 41.7 ± 0.71 μM, respectively. Compound **4** exhibited cytotoxicity against Hela and BEL-7042 cell lines with IC_50_ values of 14.9 ± 0.21 μM and 26.7 ± 1.1 μM, respectively. In addition, all of the compounds were tested for their inhibitory activity against α-glycosidase (Table 3). Only compounds **4** and **5** displayed the inhibitory activity against α-glycosidase, with IC_50_ values of 78.2 ± 2.1 μM and 49.3 ± 10.6 μM, respectively.

## 3. Materials and Methods

### 3.1. General Experimental Procedures

Silica gel (60–80, 200–300 mesh, Qingdao Marine Chemical Co. Ltd., Qingdao, China), ODS gel (20–45 μm, Fuji Silysia Chemical Co. Ltd., Aichi-ken, Japan,), and Sephadex LH-20 (Merck, Kenilworth, NJ, USA) were used for column chromatography. TLC was conducted on precoated silica gel G plates (Qingdao Marine Chemical Co. Ltd., Qingdao, China), and spots were detected by spraying with 5% H_2_SO_4_ in EtOH followed by heating. Optical rotations were measured on a Rudolph Autopol III polarimeter (Rudolph Research Analytical, NJ, USA). IR absorptions were obtained on a Nicolet 380 FT-IR instrument (Thermo, Waltham, MA, USA) using KBr pellets. 1D and 2D NMR spectra were recorded on Bruker AV III spectrometer (Bruker, Billerica, MA, USA) (^1^H NMR at 500 MHz and ^13^C NMR at 125 MHz for **2**–**5**, ^1^H NMR at 600 MHz and ^13^C NMR at 150 MHz for compound **1**) using TMS as the internal standard. HRESIMS spectra were recorded with Agilent 6200/6500 iFunnel Q-TOF. Semipreparative HPLC was carried out using an ODS column (YMC-pack ODS-A, 10 × 250 mm, 5*μ*m, 4 mL/min). Chem3D Pro 14.0 was the software used for building these 3D models and the calculation method used for energy minimizations. 

### 3.2. Fungal Material

The strain HNWSW-1 of *Cladosporium* sp. was isolated from the healthy tree root of *Ceriops tagal*, which was collected in the Dong Zhai Gang Mangrove Reserve in Hainan province in July 2011. A healthy root sample of *Ceriops tagal* was washed in running tap water to remove adhered epiphytes and soil debris. The root surface was sterilized by sequential immersion in 75% (*v*/*v*) ethanol for 3 min and 5% sodium hypochlorite solution for 5 min after drying under sterile conditions, washing the surface-treated roots three times in sterile distilled water after each sterilization. Next, air-dried sterilized roots were cut into about 0.5 cm × 0.5 cm cubes, deposited on a Potato Dextrose Agar (PDA) (200 g potato, 20 g glucose, 20 g agar per liter of seawater collected in Haikou Bay, China) plate containing chloramphenicol (100 μg/mL) as a bacterial inhibitor. A reference culture is maintained in our laboratory at −80 °C. Working stocks were prepared on PDA slants stored at 4 °C.

The fungus was identified based on the DNA sequences (GenBank access No. MH 535968, the the 18S rRNA gene sequences data in Supplementary Materials) of 18Sr DNA genes. The mycelium was ground to a fine powder in liquid N_2_, then genomic DNA was extracted, and the 18S rDNA region was amplified by PCR using primers NS1 (5′-GTAG TCATATGCTTGTCTC-3′) and NS6 (5′-GCATCACAGACCTGTTATTGCCT C-3′). PCR products were sequenced (Applied Biosystems 3730 XL Genetic Analyzer, Applied Biosystems Inc., Foster City, CA, USA).

### 3.3. Fermentation and Extraction

*Cladosporium* sp. HNWSW-1 was cultured in PDB (the potato liquid media consisting of 200.0 g/L potato, 20.0 g/L glucose, and 1000 mL deionized water) at 28 °C and 150 rpm for 72 h. Then 5 mL seed broth was transferred aseptically to 1000 ml Erlenmeyer flasks (60 flasks), each containing rice medium (80 g rice and 160 mL water). The flasks were incubated at room temperature under static conditions for 60 days. The cultures were extracted three times by EtOAc, and the EtOAc solutions were combined and evaporated under reduced pressure to produce a dark brown gum (7.1 g).

### 3.4. Purification and Identification

The obtained EtOAc crude extract (7.1 g) was fractionated into 12 fractions (Fr.1–Fr.12) on silica gel VLC and eluted with a gradient elution of CH_2_Cl_2_-petroleum ether (0–100%) and MeOH-CH_2_Cl_2_ (0–100%). Fr.6 (2.4 g) was fractionated by RP-18 column chromatography with a gradient of water-MeOH to give 25 fractions (Fr.6.1–Fr.6.25). Fr.6.9 (118.2 mg) was purified by a sephadex LH-20 column (40 g) and eluted with MeOH and acetone, respectively, then further submitted to HPLC purification on ODS column eluting with 90% MeOH to yield compounds **1** (1.0 mg) and **2** (2.1 mg). Fr.6.10 (72.6 mg) was separated by a sephadex LH-20 column (40 g) and eluted with MeOH to yield **4** (2.0 mg). Fr.8 (853.1 mg) was submitted to an RP-18 column and eluted with MeOH-Water to give 7 fractions (Fr.8.1–Fr.8.7). Fr.8.2 (25.2 mg) was separated by a sephadex LH-20 column (15 g) and eluted with MeOH and further submitted to HPLC purification on ODS column eluted with 35% MeCN to yield **3** (0.6 mg) and **5** (2.2 mg).

Cladosporitin A (**1**): Yellow oil;${\left[\alpha \right]}_{D}^{20}$—166.6 (*c* 0.01, CHCl_3_); IR (KBr) ν_max_: 3290, 2926,1711,1515 cm^−1^; HRESIMS: *m/z* 528.3103 [M + Na]^+^ (calcd. for C_32_H_43_NO_4_Na, 528.3084); ^1^H and ^13^C NMR data: See Table 1.

Cladosporitin B (**2**): Yellow oil; ${\left[\alpha \right]}_{D}^{20}$—94.5 (*c* 0.11, CHCl_3_); IR (KBr) ν_max_: 3247, 2956, 1713, 1515 cm^−1^; HRESIMS: *m*/*z* 506.3279 [M + H]^+^ (calcd. for C_32_H_44_NO_4_, 506.3265); ^1^H and ^13^C NMR data: See Table 1.

Clapone (**3**): Yellow oil; IR (KBr) ν_max_: 3435, 2978, 1673, 1400 cm^−1^; HRESIMS: *m*/*z* 239.0676 [M + Na]^+^ (calcd. for C_1__3_H_12_O_3_Na, 239.0679); ^1^H and ^13^C NMR data: See Table 2.

### 3.5. Bioassays for Cytotoxic Activity

The MTT method optimized by Mosmann et al. [26] was performed in vitro to test the cytotoxic activity of compounds 1–6. Adriamycin was used as a positive control and the medium without the test compound was used as a negative control in the bioassay. 

### 3.6. Bioassays for α-Glycosidase Inhibitory Activity

The method optimized by Jong et al. [27] was performed in vitro to test the α-glucosidase inhibitory activity of compounds 1–5. Acarbose was used as positive control.

## 4. Conclusions

Three new compounds (**1**–**3**) along with the previously reported talaroconvolutin A (**4**) and anthraquinone (**5**) were isolated from the rice medium culture of mangrove-derived fungus *Cladosporium* sp. HNWSW-1, isolated from the healthy root of *Ceriops tagal* collected in the Dong Zhai Gang Mangrove Reserve in Hainan. Their structures were determined by spectroscopic methods. Compound **2** showed cytotoxicity against BEL-7042, K562 and SGC-7901 cell lines with IC_50_ values of 29.4 ± 0.35 μM, 25.6 ± 0.47 μM, and 41.7 ± 0.71 μM, respectively, while compound **4** exhibited cytotoxicity against the Hela and BEL-7042 cell lines with IC_50_ values of 14.9 ± 0.21 μM and 26.7 ± 1.1 μM, respectively. Moreover, compounds **4** and **5** displayed inhibitory activity against α-glycosidase with IC_50_ values of 78.2 ± 2.1 μM and 49.3 ± 10.6 μM, respectively. The results suggested that the mangrove-derived fungi are an important source of new bioactive substances.

## Figures and Tables

**Table 1:** ^1^H and ^13^C NMR Data for compound **1** (600 and 150 MHz, δ in ppm) and compound **2** (500 and 125 MHz, δ in ppm).

Position	1 ^a^		2 ^b^	
	*δ*_C__,_ Type	*δ*_H__,_ Mult. (*J* in Hz)	*δ*_C_, Type	*δ*_H__,_ mult. (*J* in Hz)
1	-	-	-	-
2	172.7, C	-	172.0, C	-
3	61.7, CH	4.06, d, (2.4)	61.9, CH	3.40, d, (4.1)
4	45.0, CH	3.40, m	43.6, CH	3.67, m
5	179.2, C		178.3, C	-
6	35.8, CH_2_	3.11, dd, (14.7, 5.9) 2.87, dd, (14.7, 5.3)	35.0, CH_2_	3.10, dd, (13.7, 5.1) 2.79, dd, (13.7, 8.7)
7	128.1, C	-	128.2, C	-
8	131.5, CH	7.10, d, (8.3)	130.5, CH	6.99, d, (8.7)
9	116.3, CH	6.77, d, (8.3)	115.9, CH	6.75 d, (8.7)
10	157.5, C	-	155.4, C	-
11	116.3, CH	6.77, d (8.3)	115.9, CH	6.75, d, (8.7)
12	131.5, CH	7.10, d (8.3)	130.5, CH	6.99, d, (8.7)
13	203.9, C	-	202.2, C	-
14	52.9, CH	3.62, dd (12.0, 7.0)	52.6, C	3.33, dd, (12.1, 6.9)
15	51.8, CH	3.10 m	50.9, CH	2.95, d, (6.9)
16	130.7, C	-	130.1, C	-
17	137.1, CH	5.38, s	135.8, CH	5.29, s
18	49.1, CH_2_	1.48, m 0.87, m	48.3, CH_2_	1.43, m 0.86, m
19	28.1, CH	1.66, m	27.4, CH	1.61, m
20	36.5, CH_2_	1.64, m 0.82, m	35.6, CH_2_	1.60, m 0.77, m
21	24.1, CH_2_	1.32, m 0.96, m	24.3, CH_2_	1.62, m 0.75, m
22	40.8, CH	1.78, ddd, (12.0, 12.0, 2.3)	42.2, CH	1.67, m
23	36.2, C	-	35.2, C	-
24	135.7, C	-	135.0, C	-
25	136.7, CH	5.05, d, (8.2)	136.1, CH	4.66, d, (9.3)
26	34.8, CH	2.25, m	33.9, CH	2.14, m
27	31.1, CH_2_	1.35, m 1.23, m	30.4, CH_2_	1.27, m 1.10, m
28	12.4, CH_3_	0.86, t, (7.6)	12.1, CH_3_	0.79, t, (7.1)
29	21.1, CH_3_	0.92, d, (6.2)	20.9, CH_3_	0.76, d, (6.6)
30	15.2, CH_3_	1.45, s	14.5, CH_3_	1.47, s
31	22.5, CH_3_	1.54, s	22.2, CH_3_	1.46, s
32	20.5, CH_3_	0.91, s	20.3, CH_3_	0.85, s
33	23.1, CH_3_	0.82, d, (6.4)	22.8, CH_3_	0.82, d, (6.2)

^a^ Recorded in Acetone; ^b^ Recorded in CDCl_3_.

**Table 2:** ^1^H and ^13^C NMR Data for compound **3** in CH_3_OD (500 and 125 MHz, *δ* in ppm).

Position	3	
	*δ*_C__,_ Type	*δ*_H__,_ Mult. (*J* in Hz)
1	-	-
2	162.0, C	-
3	109.9, CH	6.01, s
4	182.3, C	-
4a	115.8, C	-
5	143.6, C	-
6	118.3, CH	6.64, dd, (2.2, 0.8)
7	164.0, C	-
8	101.8, CH	6.70, d, (2.2)
8a	161.0, C	-
1′	124.8, CH	6.26, ddd, (15.6, 3.4, 1.7)
2′	137.3, CH	6.86, ddd, (15.6, 13.7, 6.9)
3′	18.6, CH_3_	1.99, dd, (6.9,1.7)
5- CH_3_	23.1, CH_3_	2.73, s

**Table 3:** Cytotoxic and α-glycosidase inhibitory activities of **1**–**5**.

Compounds	IC50 (μM)				
	Hela	BEL-7042	K562	SGC-7901	α-Glycosidase
1	>50	>50	>50	>50	>500
2	>50	29.4 ± 0.35	25.6 ± 0.47	41.7 ± 0.71	>500
3	>50	>50	>50	>50	>500
4	14.9 ± 0.21	26.7 ± 1.1	>50	>50	78.2 ± 2.1
5	>50	>50	>50	>50	49.3 ± 10.6
Adriamycin	11.5 ± 0.18	11.9 ± 0.37	14.2 ± 0.66	6.66 ± 0.2	ND ^a^
Acarbose	ND ^a^	ND ^a^	ND ^a^	ND ^a^	275.7 ± 2.7

^a^ Not detected; Values are expressed as mean ± standard deviation (SD); *n* = 3.

## Supplementary Materials

The NMR and HRESIMS spectra for **1**–**3** are available online at http://www.mdpi.com/1660-3397/17/1/4/s1.
