# Supplementary material for: Two New Succinimide Derivatives Cladosporitins A and B from the Mangrove-derived Fungus Cladosporium sp. HNWSW-1
Source: Mar Drugs. 2018 Dec 20;17(1):4. doi: 10.3390/md17010004 (PMC6356855; doi:10.3390/md17010004)
Supplement: Supplementary file 1 [file marinedrugs-17-00004-s001.pdf]

Supplementary Information

Table of Contents

The 18S rRNA gene sequences data of *Cladosporium* sp. HNWSW-1

Figure S1. The ^{1}H NMR (600 MHz, CD_3COCD_3-d_6) spectrum of compound **1**

Figure S2. The DEPTQ (150 MHz, CD_3COCD_3-d_6) spectrum of compound **1**

Figure S3. The ^{1}H-^{1}H COSY (600 MHz, CD_3COCD_3-d_6) spectrum of compound **1**

Figure S4. Slice 1 of ^{1}H-^{1}H COSY (600 MHz, CD_3COCD_3-d_6) spectrum of compound **1**

Figure S5. Slice 2 of ^{1}H-^{1}H COSY (600 MHz, CD_3COCD_3-d_6) spectrum of compound **1**

Figure S6. The HSQC (600 MHz, CD_3COCD_3-d_6) spectrum of compound **1**

Figure S7. Slice 1 of HSQC (600 MHz, CD_3COCD_3-d_6) spectrum of compound **1**

Figure S8. Slice 2 of HSQC (600 MHz, CD_3COCD_3-d_6) spectrum of compound **1**

Figure S9. The HMBC (600 MHz, CD_3COCD_3-d_6) spectrum of compound **1**

Figure S10. Slice 1 of HMBC (600 MHz, CD_3COCD_3-d_6) spectrum of compound **1**

Figure S11. Slice 2 of HMBC (600 MHz, CD_3COCD_3-d_6) spectrum of compound **1**

Figure S12. Slice 3 of HMBC (600 MHz, CD_3COCD_3-d_6) spectrum of compound **1**

Figure S13. Slice 4 of HMBC (600 MHz, CD_3COCD_3-d_6) spectrum of compound **1**

Figure S14. Slice 5 of HMBC (600 MHz, CD_3COCD_3-d_6) spectrum of compound **1**

Figure S15. The ROESY (600 MHz, CD_3COCD_3-d_6) spectrum of compound **1**

Figure S16. Slice 1 of ROESY (600 MHz, CD_3COCD_3-d_6) spectrum of compound **1**

Figure S17. Slice 2 of ROESY (600 MHz, CD_3COCD_3-d_6) spectrum of compound **1**

Figure S18. The HRESIMS spectrum of compound **1**

Figure S19. The ^{1}H NMR (500 MHz, $CDCl_3$-d) spectrum of compound **2**

Figure S20. The ^{13}C NMR and DEPT-135 (125 MHz, $CDCl_3$-d) spectrum of compound **2**

Figure S21. Enlarged ^{13}C NMR (125 MHz, $CDCl_3$-d) spectrum of compound **2**

Figure S22. The ^{1}H-1H COSY (500 MHz, $CDCl_3$-d) spectrum of compound **2**

Figure S23. Slice 1 of ^{1}H-1H COSY (500 MHz, $CDCl_3$-d) spectrum of compound **2**

Figure S24. Slice 2 of ^{1}H-1H COSY (500 MHz, $CDCl_3$-*d*) spectrum of compound **2**

Figure S25. Slice 3 of ^{1}H-1H COSY (500 MHz, $CDCl_3$-*d*) spectrum of compound **2**

Figure S26. The HSQC (500 MHz, $CDCl_3$-*d*) spectrum of compound **2**

Figure S27. Slice 1 of the HSQC (500 MHz, $CDCl_3$-*d*) spectrum of compound **2**

Figure S28. Slice2 of the HSQC (500 MHz, $CDCl_3$-*d*) spectrum of compound **2**

Figure S29. Slice 3 of the HSQC (500 MHz, $CDCl_3$-*d*) spectrum of compound **2**

Figure S30. The HMBC (500 MHz, $CDCl_3$-*d*) spectrum of compound **2**

Figure S31. Slice 1 of the HMBC (500 MHz, $CDCl_3$-*d*) spectrum of compound **2**

Figure S32. Slice 2 of the HMBC (500 MHz, $CDCl_3$-*d*) spectrum of compound **2**

Figure S33. Slice 3 of the HMBC (500 MHz, $CDCl_3$-*d*) spectrum of compound **2**

Figure S34. Slice 4 of the HMBC (500 MHz, $CDCl_3$-*d*) spectrum of compound **2**

Figure S35. Slice 5 of the HMBC (500 MHz, $CDCl_3$-*d*) spectrum of compound **2**

Figure S36. Slice 6 of the HMBC (500 MHz, $CDCl_3$-*d*) spectrum of compound **2**

Figure S37. The ROESY (500 MHz, $CDCl_3$-*d*) spectrum of compound **2**

Figure S38. Slice 1 of the ROESY (500 MHz, $CDCl_3$-*d*) spectrum of compound **2**

Figure S39. The HRESIMS spectrum of compound **2**

Figure S40. The ^{1}H NMR (500 MHz, CD_3OH-d_4) spectrum of compound **3**

Figure S41. Enlarged ^{1}H NMR (500 MHz, CD_3OH-d_4) spectrum of compound **3**

Figure S42. The ^{13}C NMR and DEPT-135 (125 MHz, CD_3OH-d_4) spectrum of compound **3**

Figure S43. The ^{1}H-^{1}H COSY (500 MHz, CD_3OH-d_4) spectrum of compound **3**

Figure S44. The HSQC (500 MHz, CD_3OH-d_4) spectrum of compound **3**

Figure S45. Slice 1 of the HSQC (500 MHz, CD_3OH-d_4) spectrum of compound **3**

Figure S46. Slice 2 of the HSQC (500 MHz, CD_3OH-d_4) spectrum of compound **3**

Figure S47. The HMBC (500 MHz, CD_3OH-d_4) spectrum of compound **3**

Figure S48. Slice 1 of HMBC (500 MHz, CD_3OH-d_4) spectrum of compound **3**

Figure S49. Slice 2 of HMBC (500 MHz, CD_3OH-d_4) spectrum of compound **3**

Figure S50. The HRESIMS spectrum of compound **3**

The 18S rDNA Gene Sequences Data of *Cladosporium* sp. HNWSW-1

5′-TTCCGTAGGTGAACCTGCGGAGGGATCATTACAGTGACCCCGGTCTAAC
CACCGGGATGTTCATAACCCTTTGTTGTCCGACTCTGTTGCCTCCGGGGCG
ACCCTGCCTTCGGGCGGGGGCTCCGGGTGGACACTTCAAACTCTTGCGTA
ACTTTGCAGTCTGAGTAAACTTAATTAATAAATTAAAACTTTTAACAACGGA
TCTCTTGGTTCTGGCATCGATGAAGAACGCAGCGAAATGCGATAAGTAATG
TGAATTGCAGAATTCAGTGAATCATCGAATCTTTGAACGCACATTGCGCCC
CCTGGTATTCCGGGGGGCATGCCTGTTCGAGCGTCATTTCACCACTCAAGC
CTCGCTTGGTATTGGGCAACGCGGTCCGCCGCGTGCCTCAAATCGACCGGC
TGGGTCTTCTGTCCCCTAAGCGTTGTGGAAACTATTCGCTAAAGGGTGCTC
GGGAGGCTACGCCGTAAAACAAACCCATTTCTAAGGTTGACCTCGGATCAG
GTAGGGATACCCGCTGAACTTAAGCATATCAATAAGCGGAGGA-3′

Figure S1. The ^{1}H NMR (600 MHz, CD_3COCD_3-d_6) spectrum of compound **1**

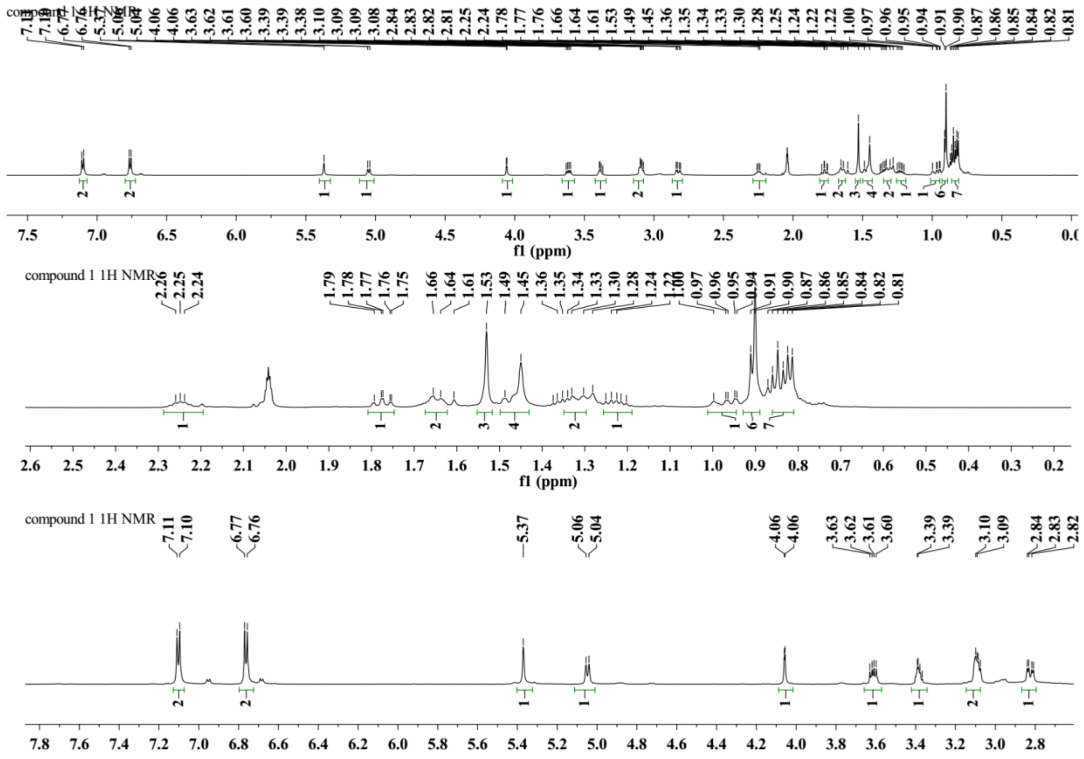

Figure S2. The DEPTQ (150 MHz, CD_3COCD_3-d_6) spectrum of compound **1**

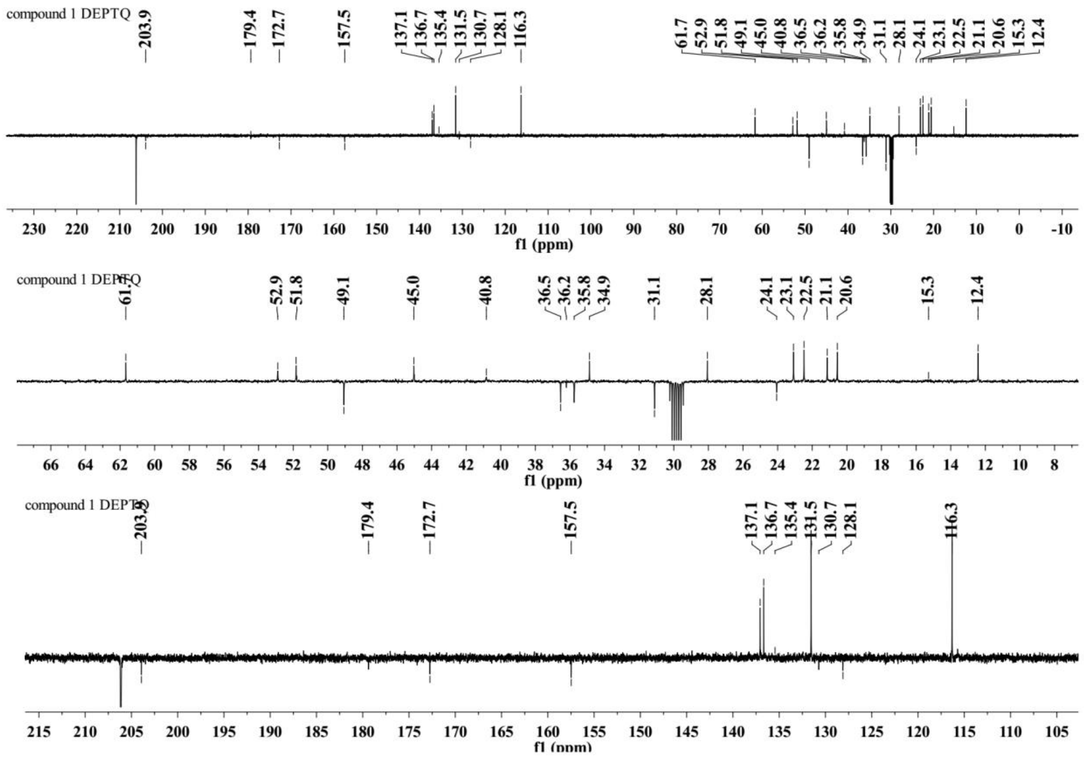

Figure S3. The ^{1}H-^{1}H COSY (600 MHz, CD_3COCD_3-d_6) spectrum of compound **1**

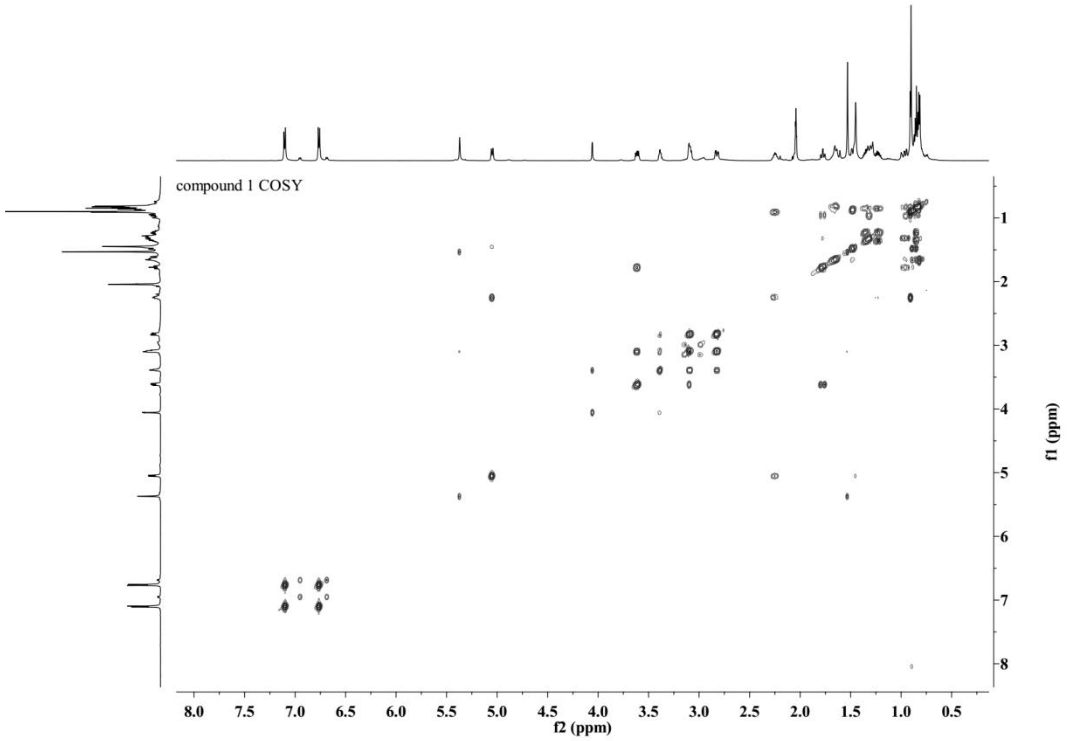

Figure S4. Slice 1 of ^{1}H-^{1}H COSY (600 MHz, CD_3COCD_3-d_6) spectrum of compound **1**

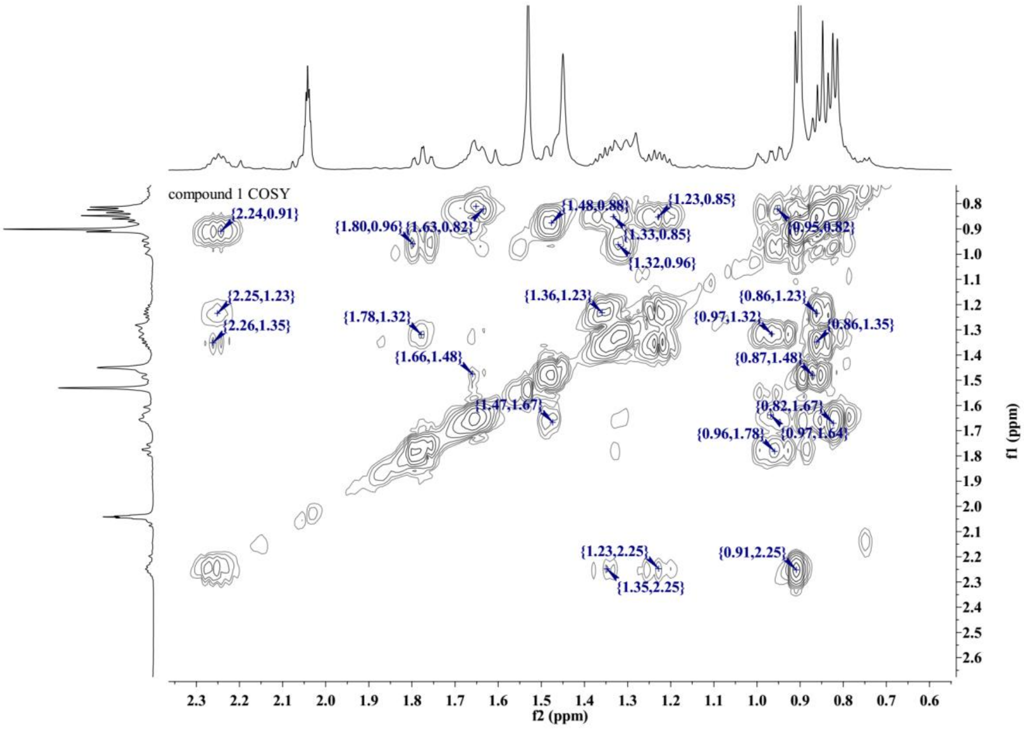

Figure S5. Slice 2 of ^{1}H-^{1}H COSY (600 MHz, CD_3COCD_3-d_6) spectrum of compound **1**

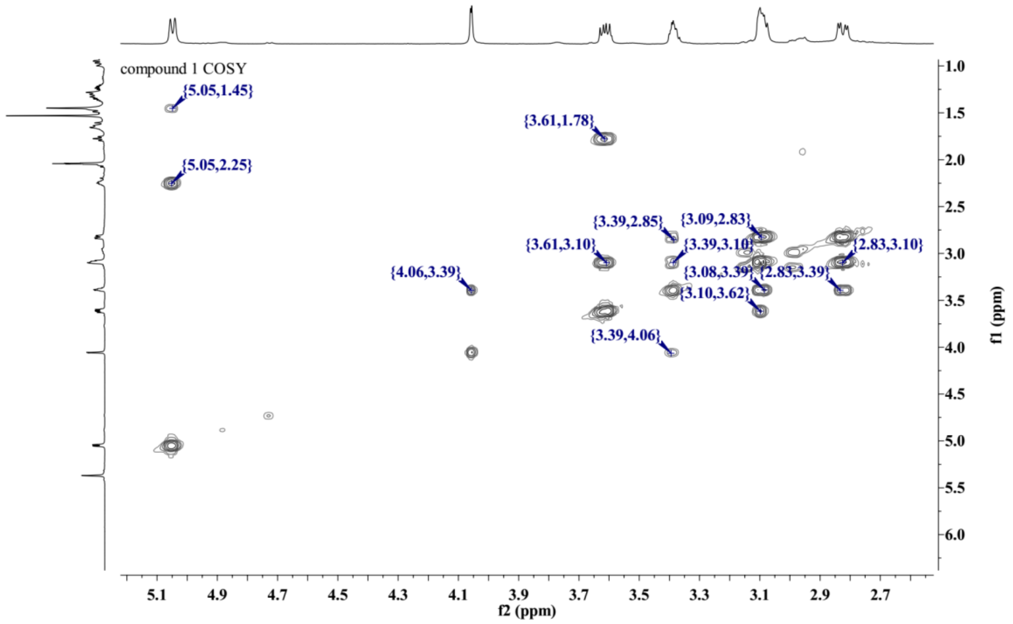

Figure S6. The HSQC (600 MHz, CD_3COCD_3-d_6) spectrum of compound **1**

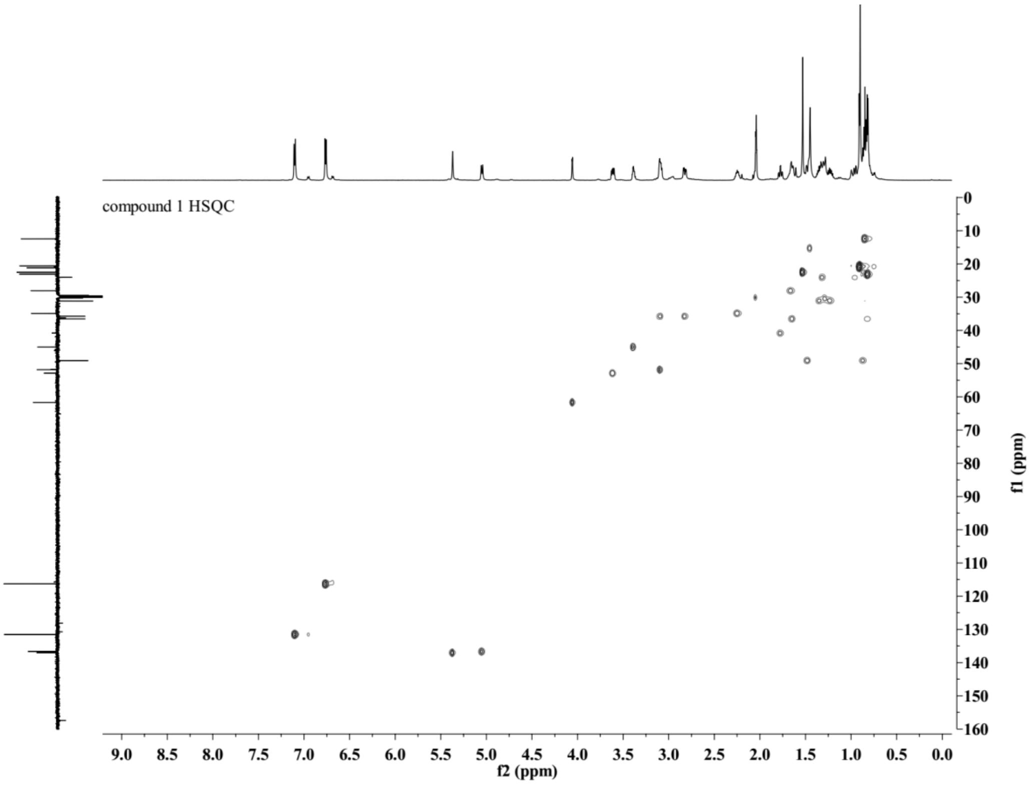

Figure S7. Slice 1 of HSQC (600 MHz, CD_3COCD_3-d_6) spectrum of compound **1**

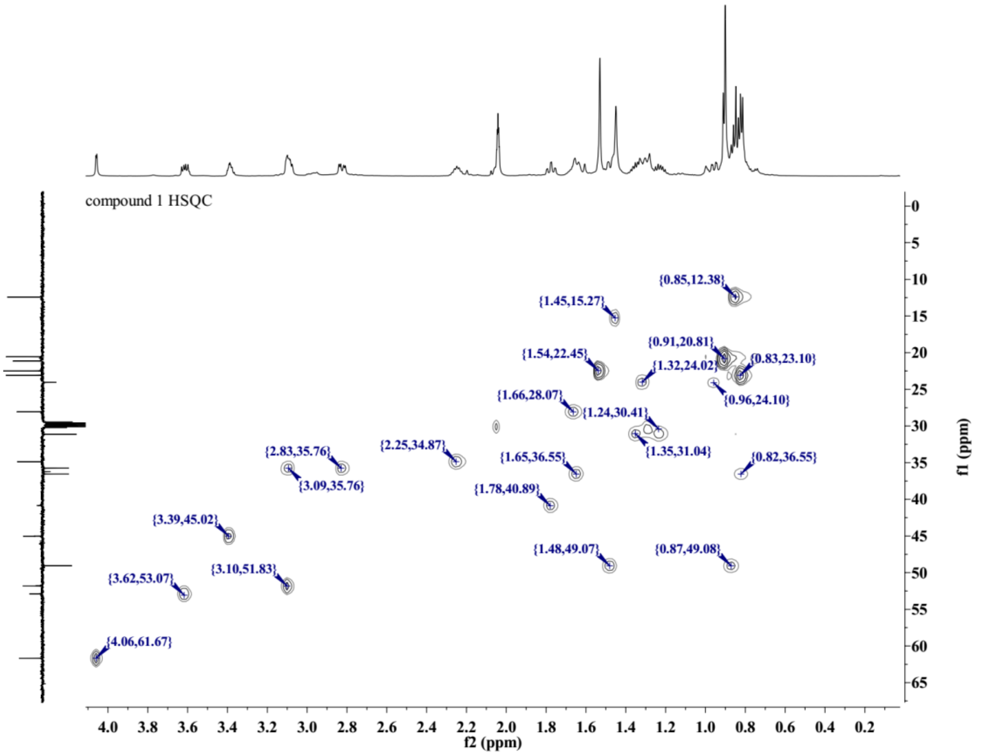

Figure S8. Slice 2 of HSQC (600 MHz, CD_3COCD_3-d_6) spectrum of compound **1**

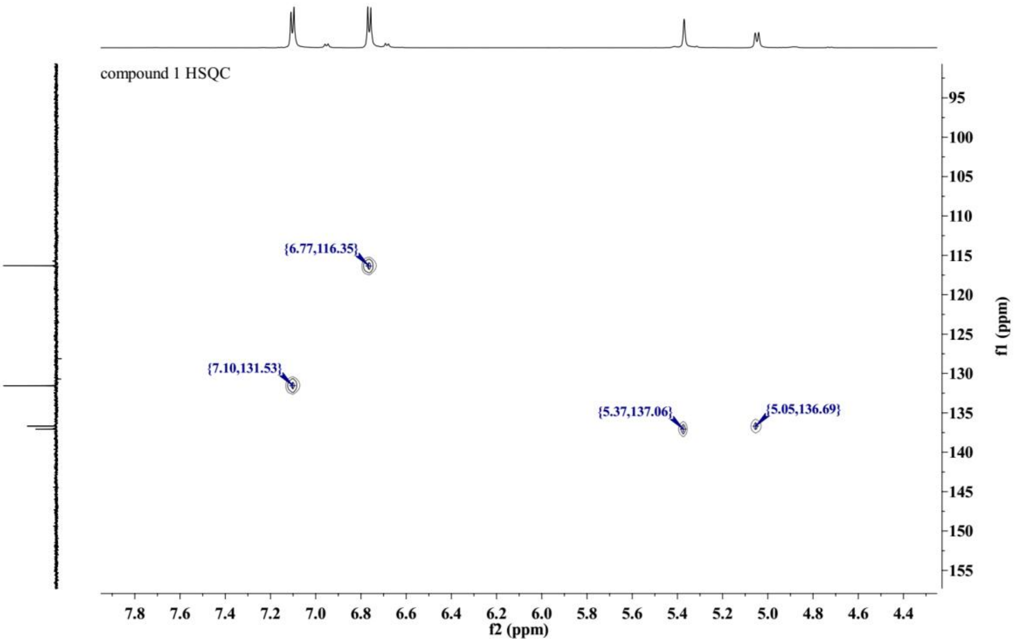

Figure S9. The HMBC (600 MHz, CD_3COCD_3-d_6) spectrum of compound **1**

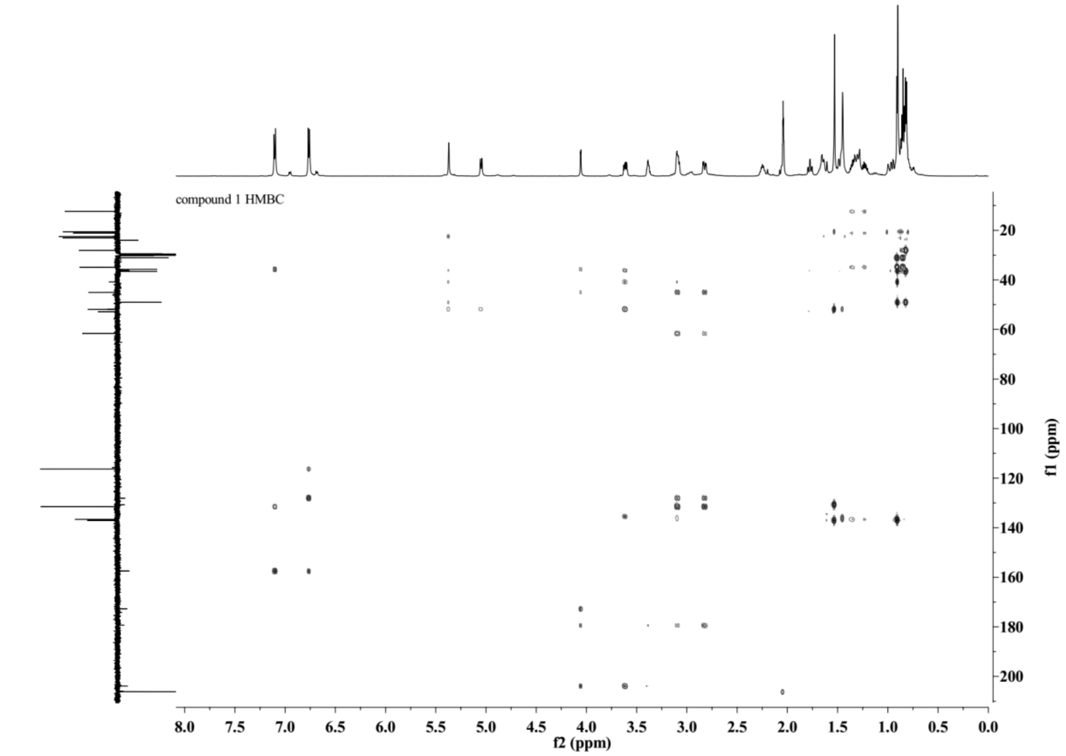

Figure S10. Slice 1 of HMBC (600 MHz, CD_3COCD_3-d_6) spectrum of compound **1**

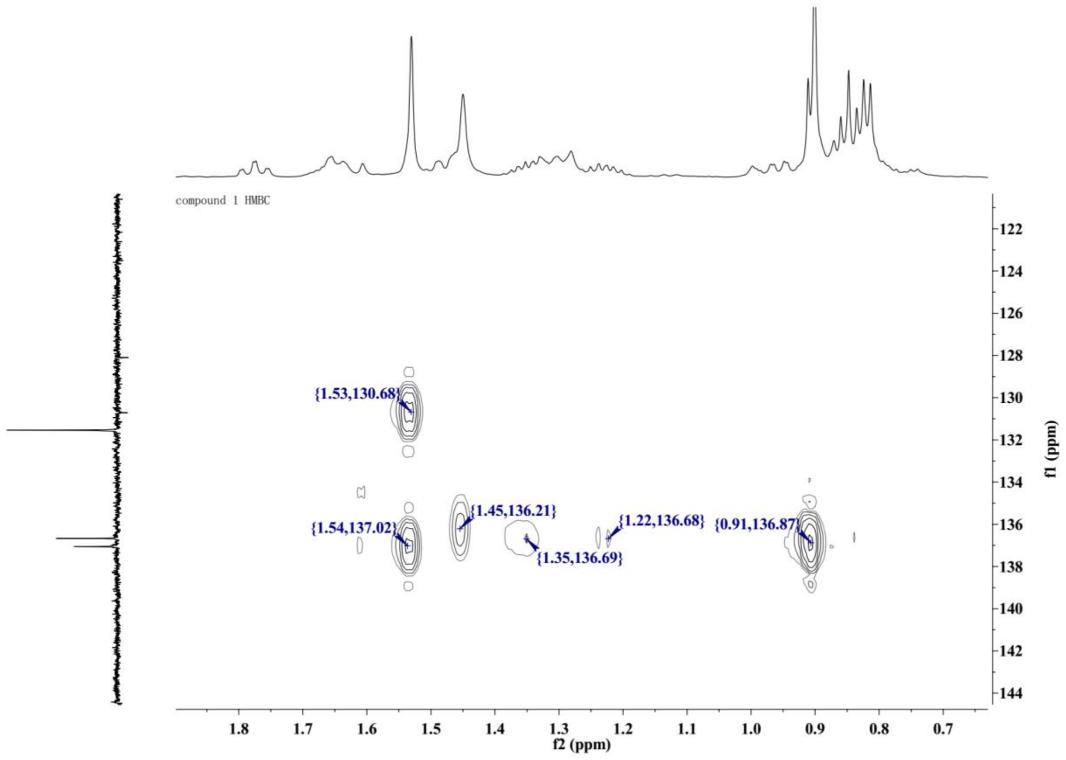

Figure S11. Slice 2 of HMBC (600 MHz, CD_3COCD_3-d_6) spectrum of compound **1**

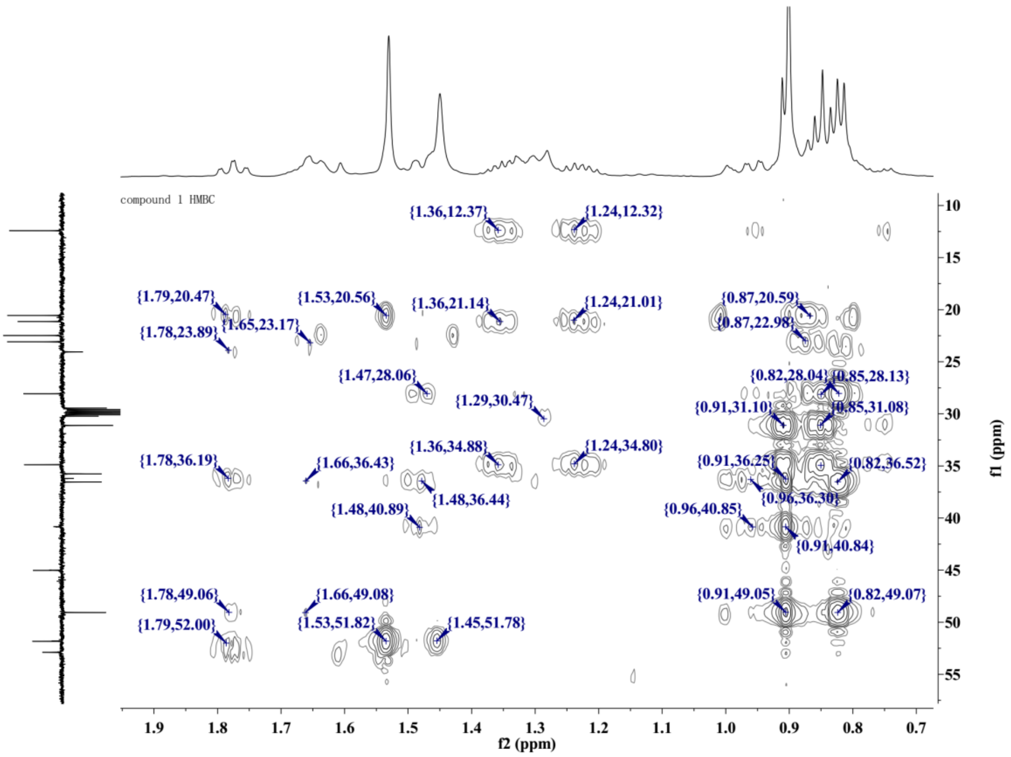

Figure S12. Slice 3 of HMBC (600 MHz, CD_3COCD_3-d_6) spectrum of compound **1**

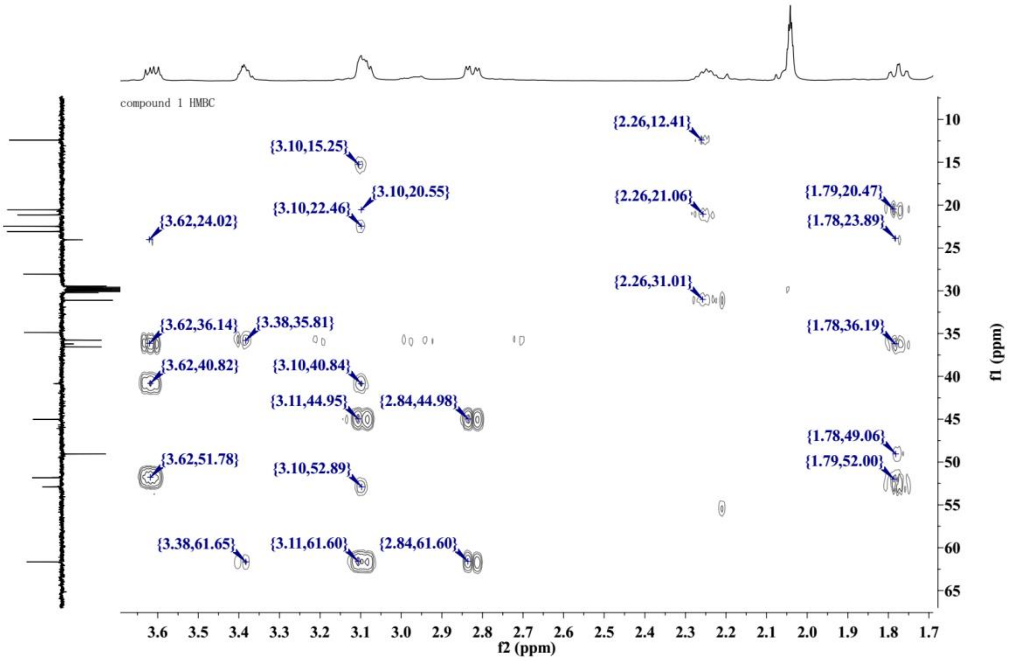

Figure S13. Slice 4 of HMBC (600 MHz, CD_3COCD_3-d_6) spectrum of compound **1**

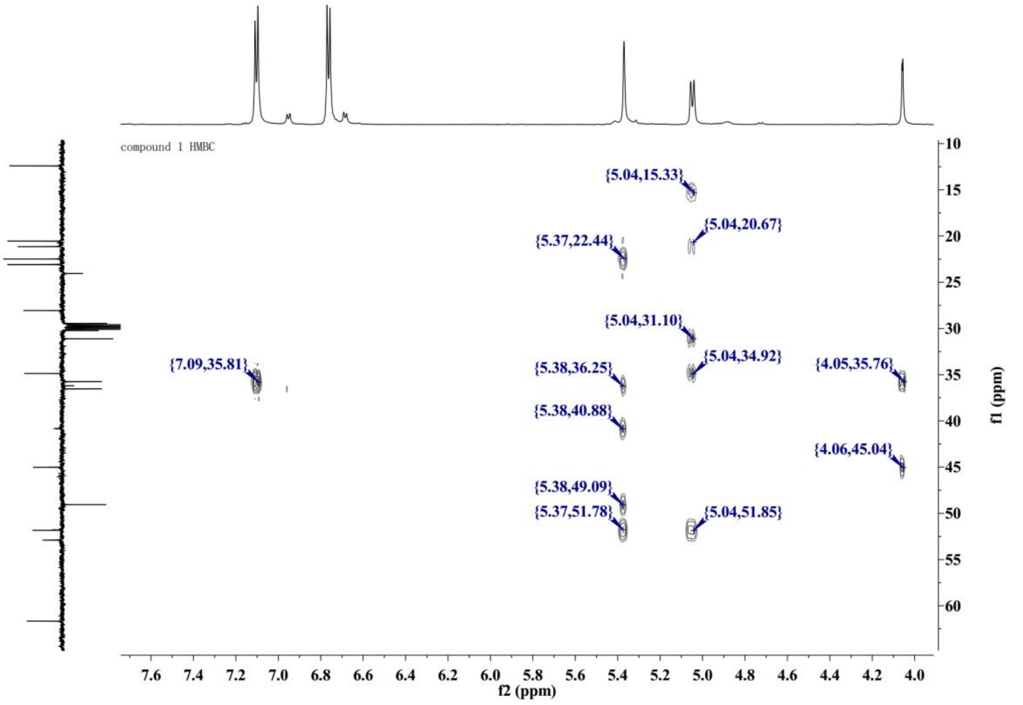

Figure S14. Slice 5 of HMBC (600 MHz, CD_3COCD_3-d_6) spectrum of compound **1**

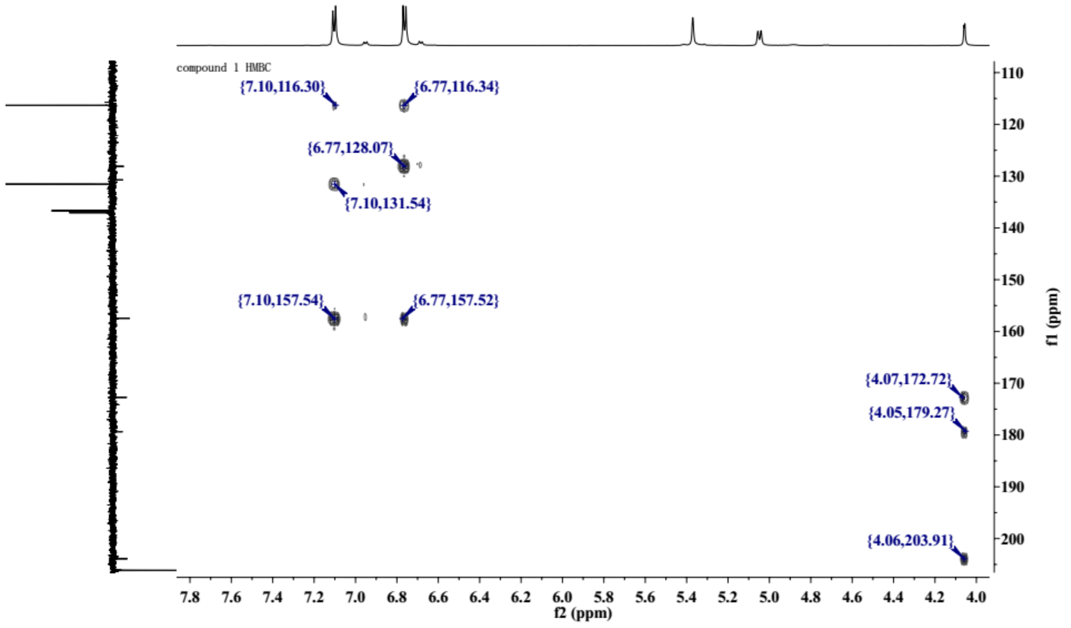

Figure S15. The ROESY (600 MHz, CD_3COCD_3-d_6) spectrum of compound **1**

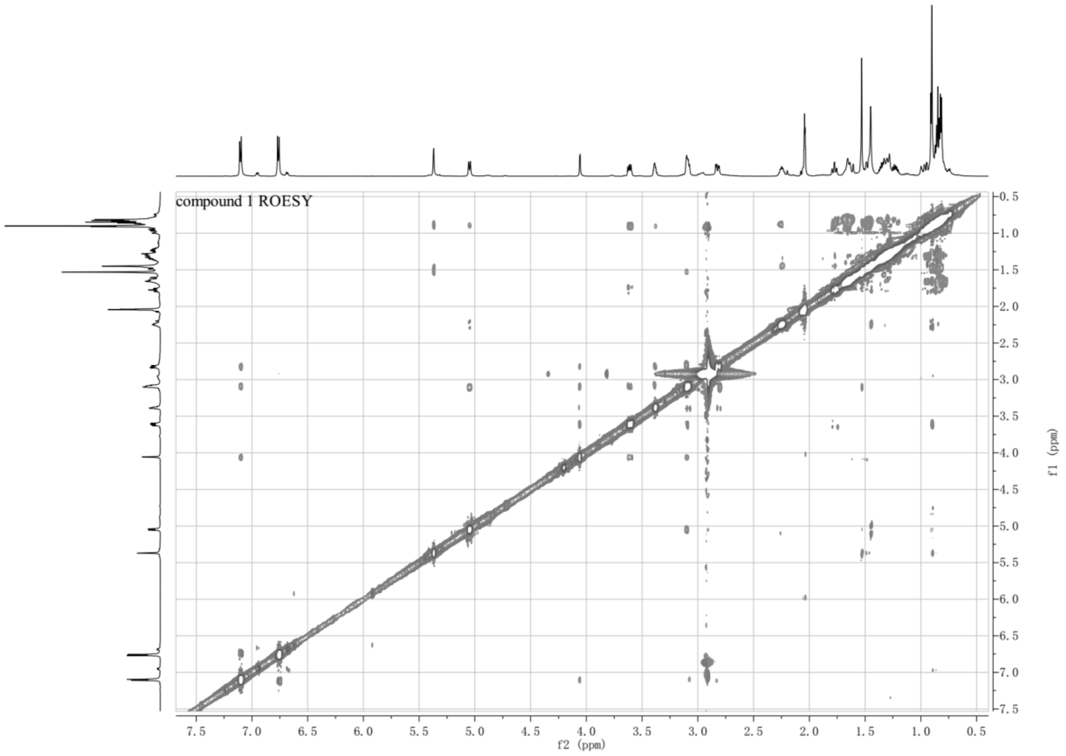

Figure S16. Slice 1 of ROESY (600 MHz, CD_3COCD_3-d_6) spectrum of compound **1**

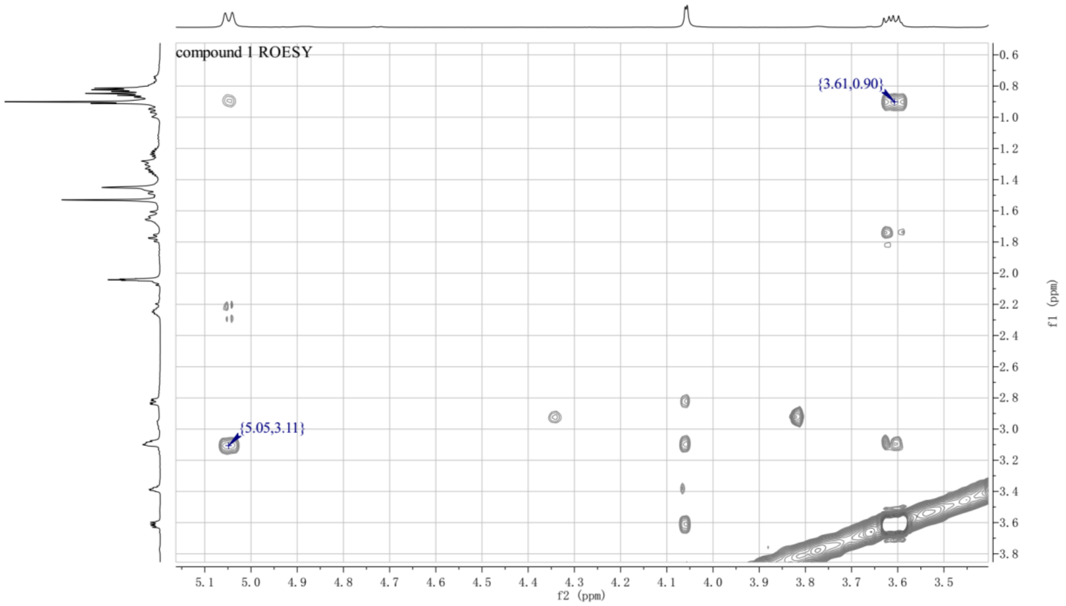

Figure S17. Slice 2 of ROESY (600 MHz, CD_3COCD_3-d_6) spectrum of compound **1**

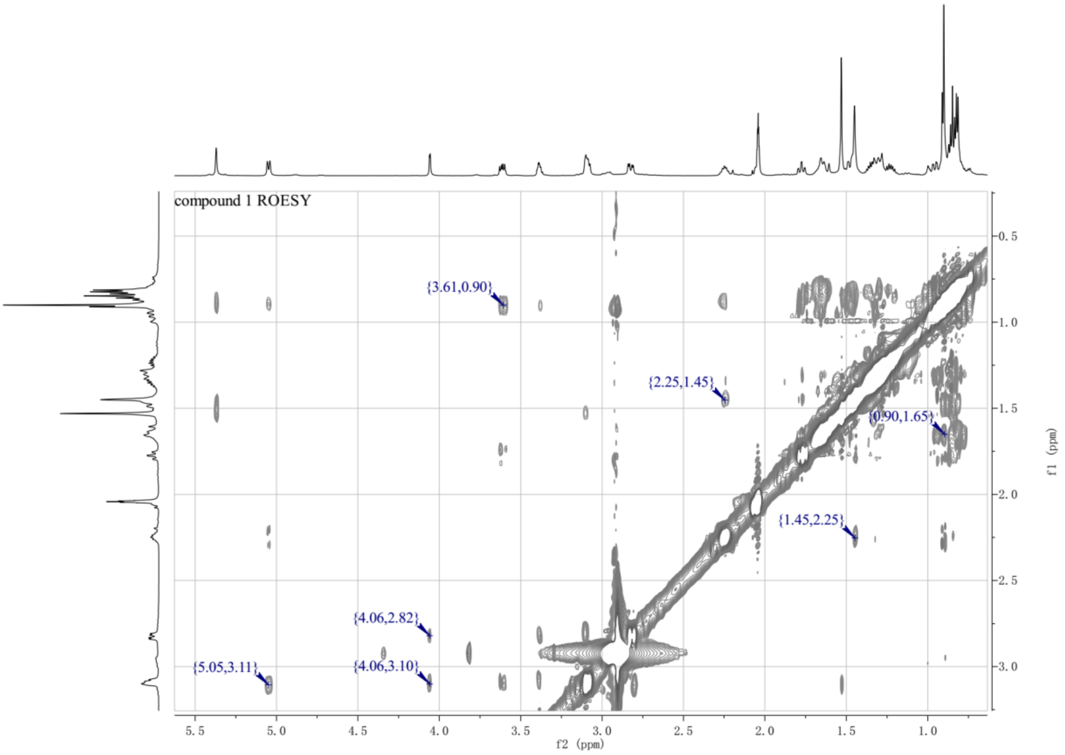

Figure S18. The HRESIMS spectrum of compound **1**

Qualitative Analysis Report

**Data Filename**	hk-dhf-10.d	**Sample Name**	hk-dhf-10
**Sample Type**	Sample	**Position**	P1-D8
**Instrument Name**	Instrument 1	**User Name**	
**Acq Method**	SIBU.m	**Acquired Time**	1/17/2017 2:41:01 PM
**IRM Calibration Status**	Success	**DA Method**	ESI+.m
**Comment**			

Sample Group **Info.**
Acquisition SW 6200 series TOF/6500 series
Version Q-TOF B.05.01 (B5125.2)

User Spectra

Fragmentor Voltage	Collision Energy	Ionization Mode
175	0	ESI

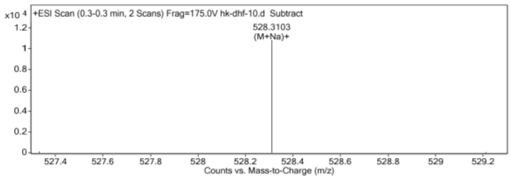

Peak List

m/z	z	Abund	Formula	Ion
168.2145	1	3420.68		
301.1435		2285.29		
506.326	1	2100.39		
528.3103	1	10759.37	C32 H43 N O4	(M+Na)+
529.3135	1	3694.58	C32 H43 N O4	(M+Na)+
544.2845	1	8712.01		
545.288	1	3143.31		
550.2912	1	2013.03		

Formula Calculator Element Limits

Element	Min	Max
C	3	60
H	0	120
O	0	30
N	0	3

Formula Calculator Results

Formula	CalculatedMass	CalculatedMz	Mz	Diff. (mDa)	Diff. (ppm)	DBE
C32 H43 N O4	505.3192	528.3084	528.3103	-1.3	-2.5	12.0000

--- End Of Report ---

Agilent Technologies Page 1 of 1 Printed at: 3:54 PM on: 1/17/2017

Figure S19. The ^{1}H NMR (500 MHz, $CDCl_3$-*d*) spectrum of compound **2**

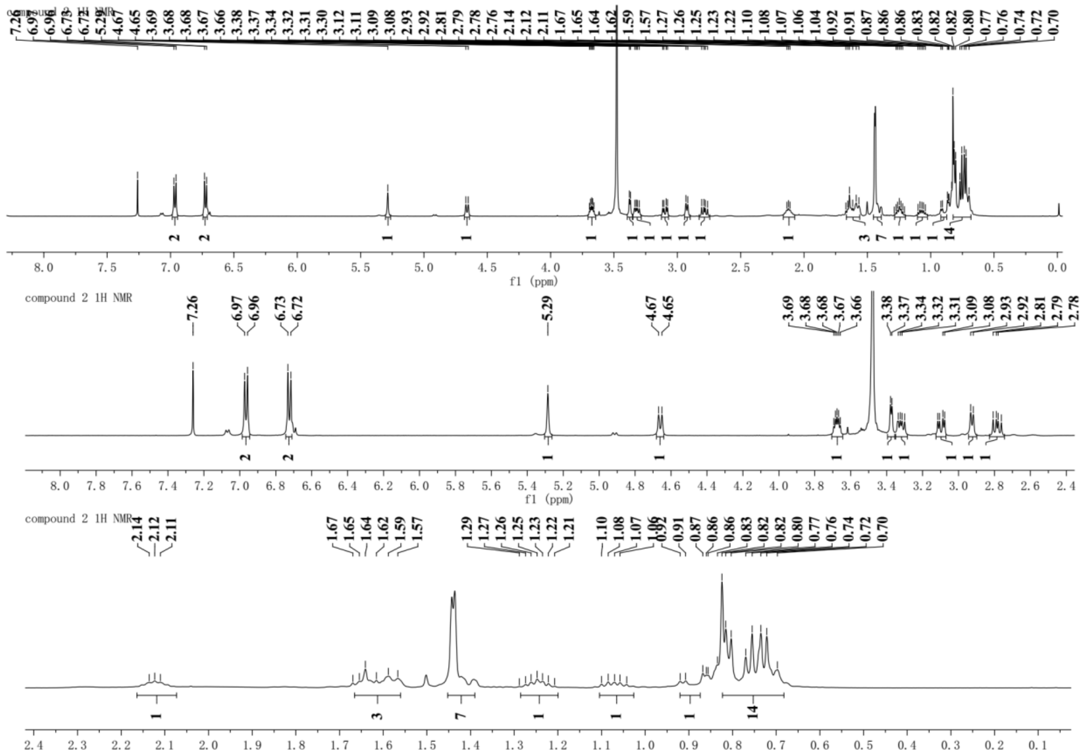

Figure S20. The ^{13}C NMR and DEPT-135 (125 MHz, $CDCl_3$-*d*) spectrum of compound **2**

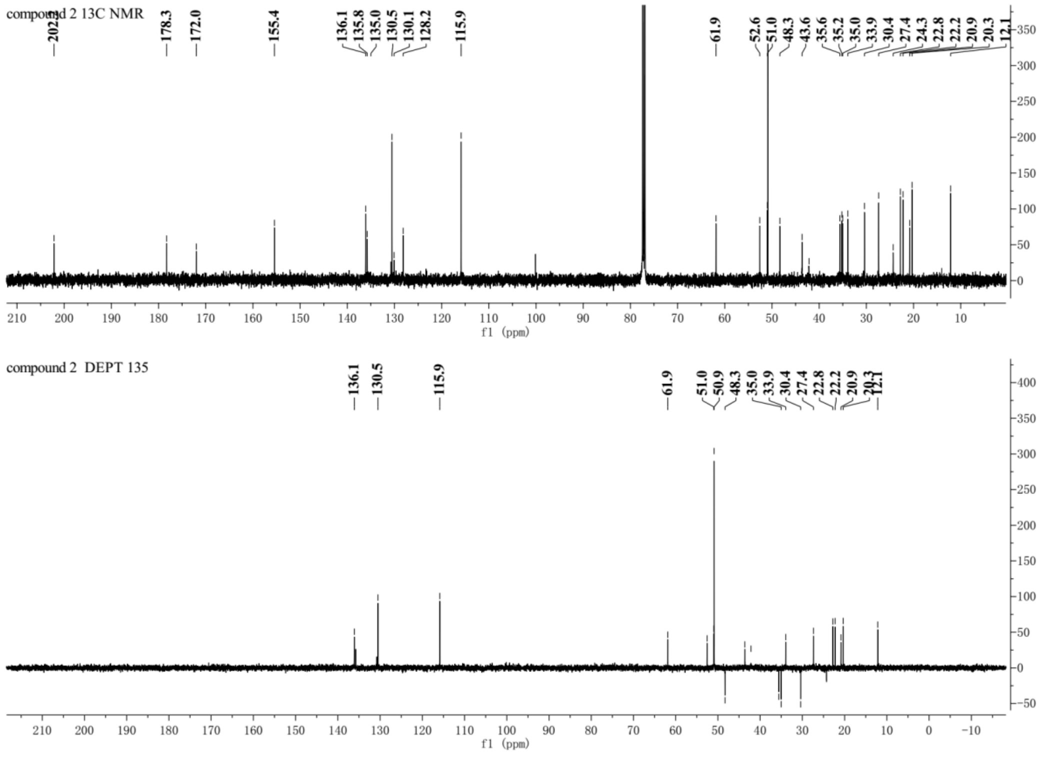

Figure S21. Enlarged ^{13}C NMR (125 MHz, $CDCl_3$-*d*) spectrum of compound **2**

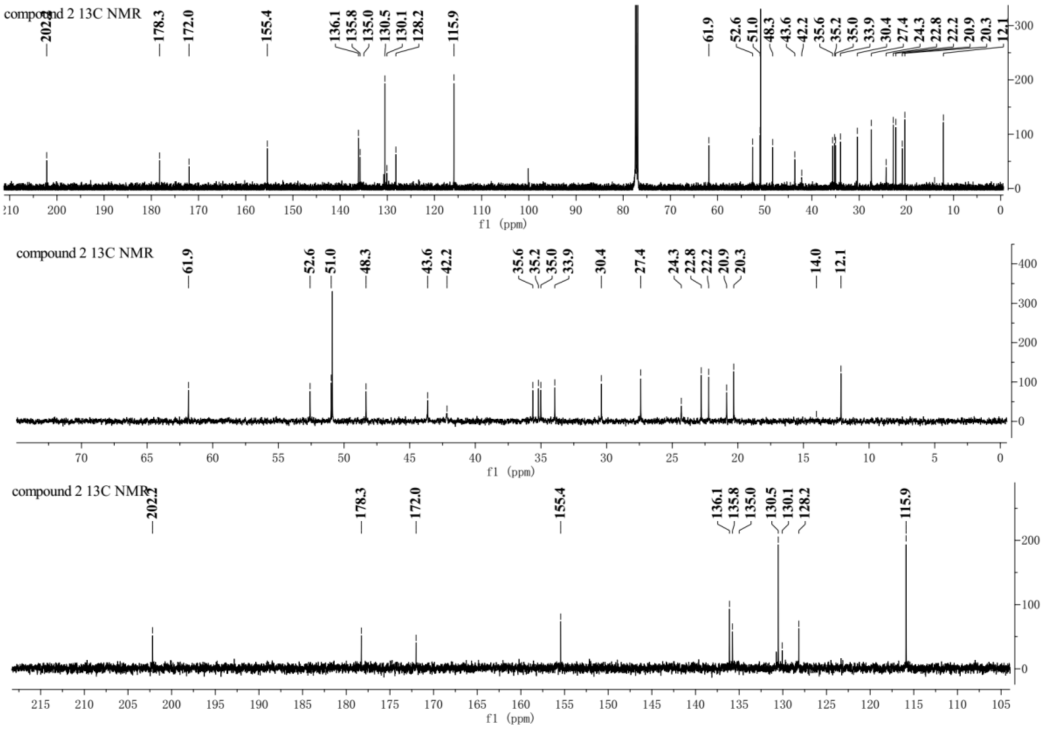

Figure S22. The ^{1}H-1H COSY (500 MHz, $CDCl_3$-*d*) spectrum of compound **2**

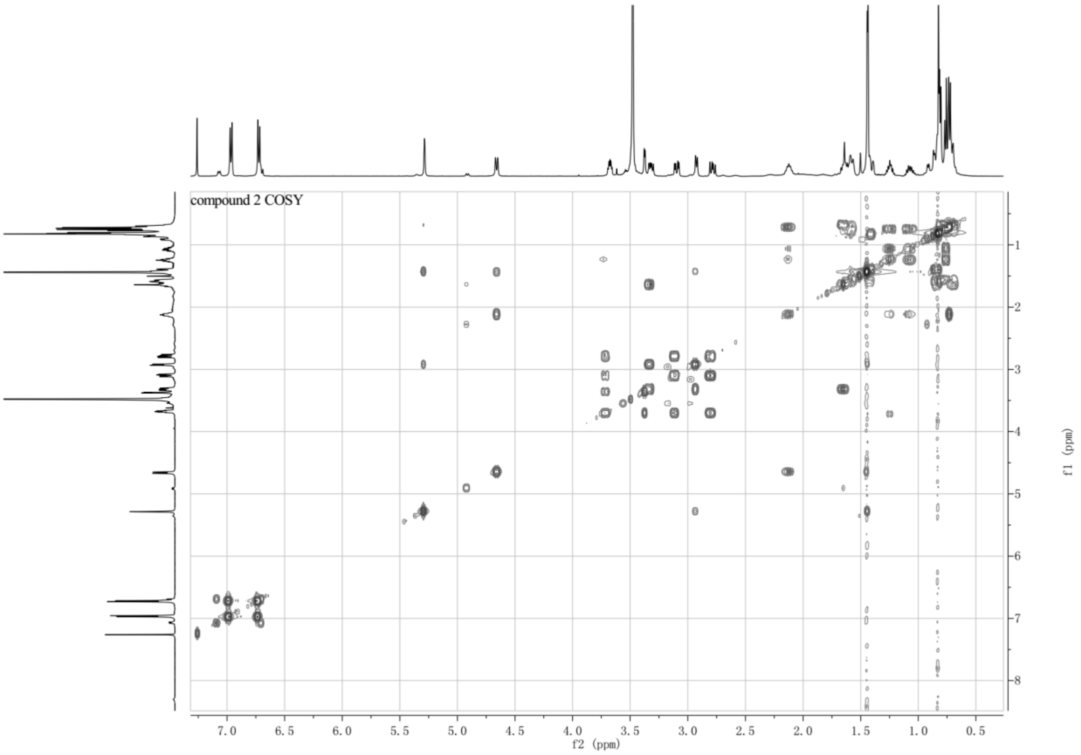

Figure S23. Slice 1 of ^{1}H-1H COSY (500 MHz, $CDCl_3$-*d*) spectrum of compound **2**

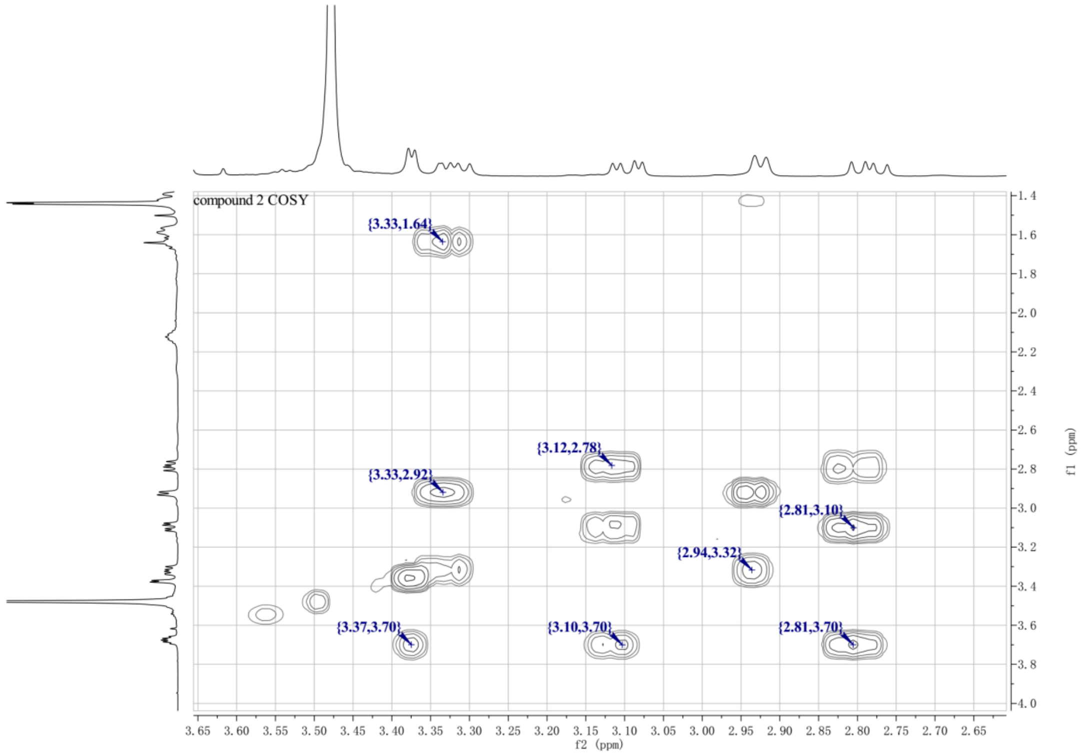

Figure S24. Slice 2 of ^{1}H-1H COSY (500 MHz, $CDCl_3$-*d*) spectrum of compound **2**

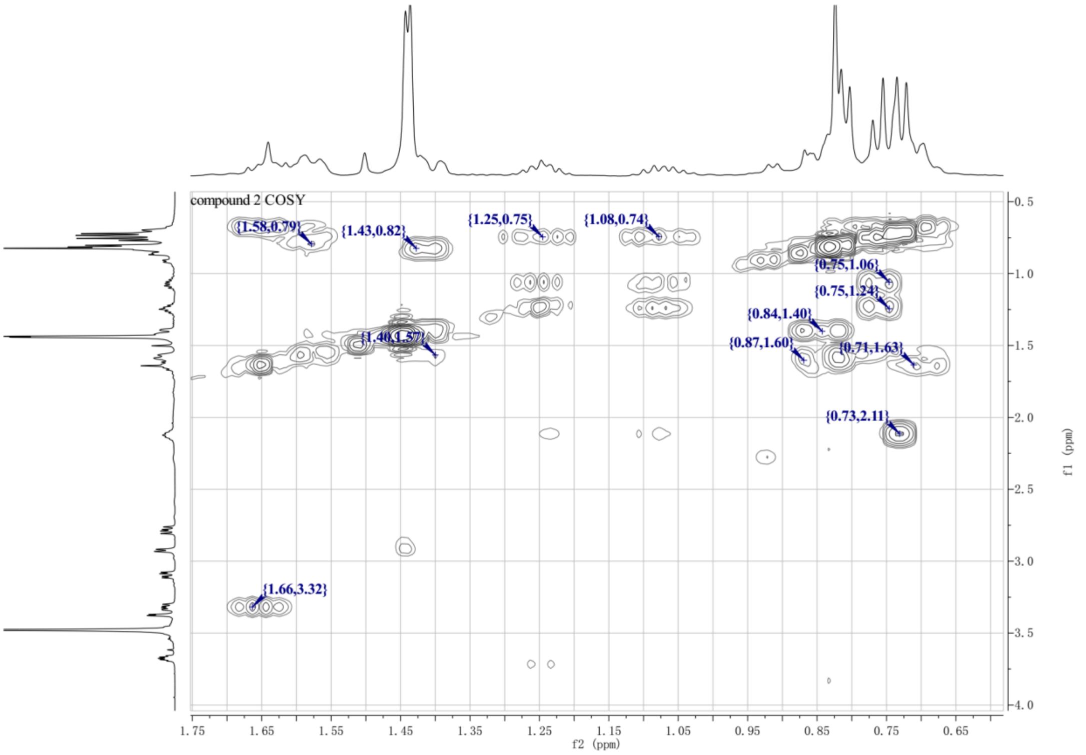

Figure S25. Slice 3 of ^{1}H-1H COSY (500 MHz, $CDCl_3$-*d*) spectrum of compound **2**

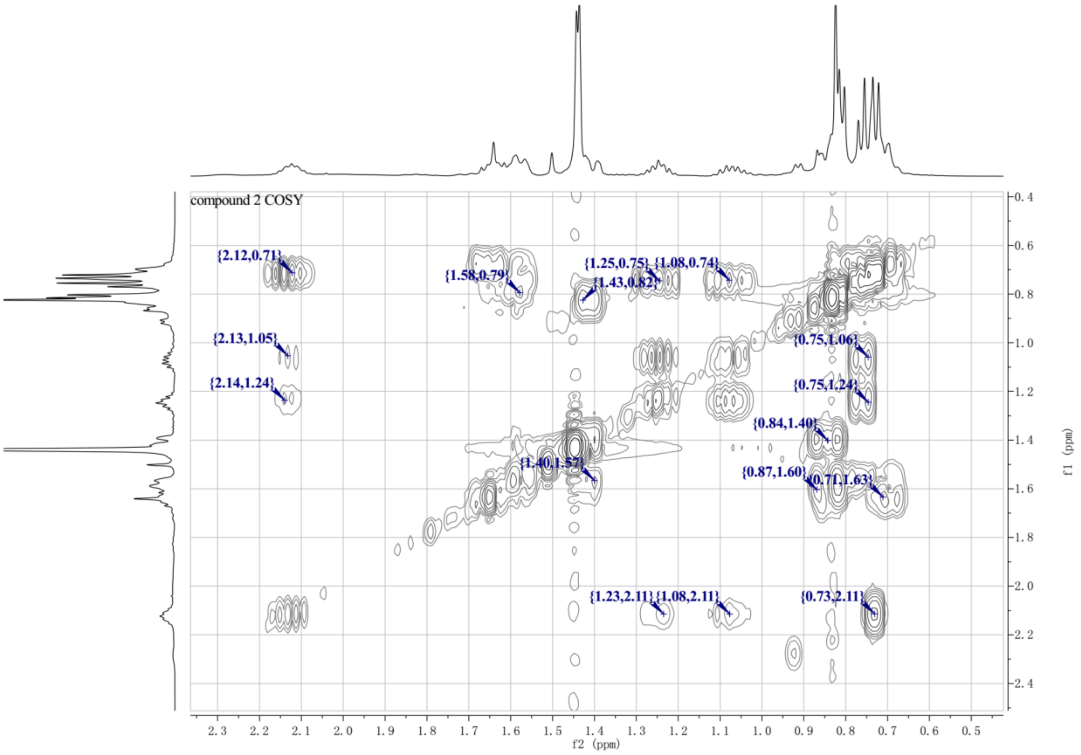

Figure S26 The HSQC (500 MHz, $CDCl_3$-*d*) spectrum of compound **2**

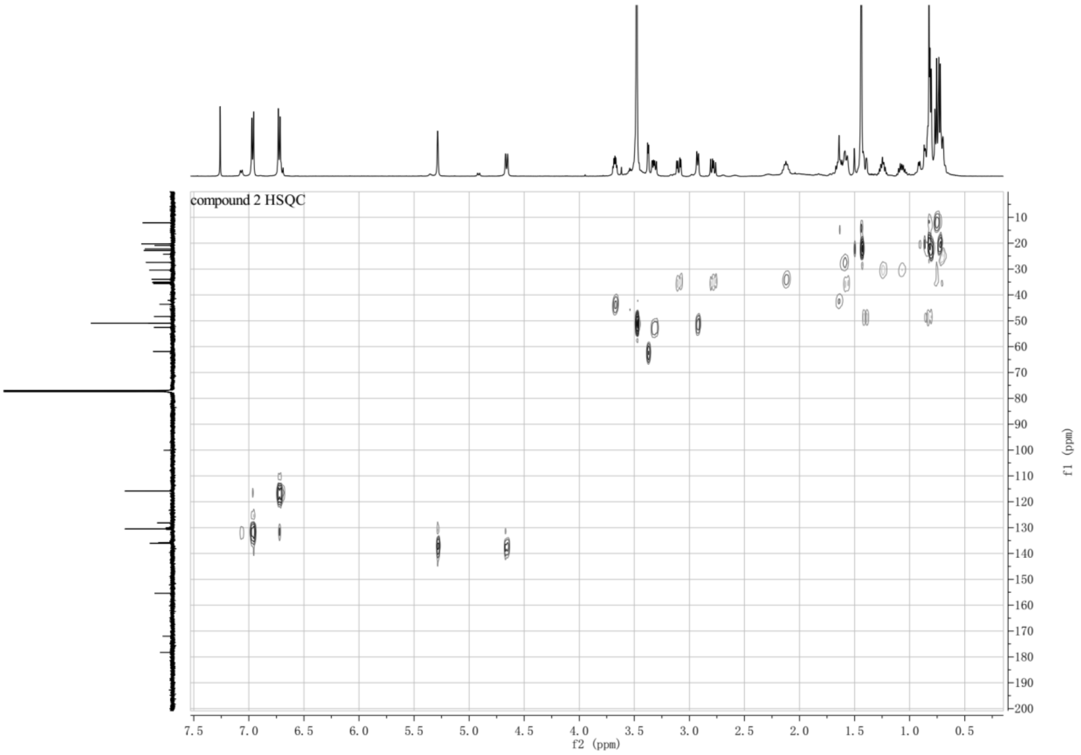

Figure S27. Slice 1 of the HSQC (500 MHz, $CDCl_3$-*d*) spectrum of compound **2**

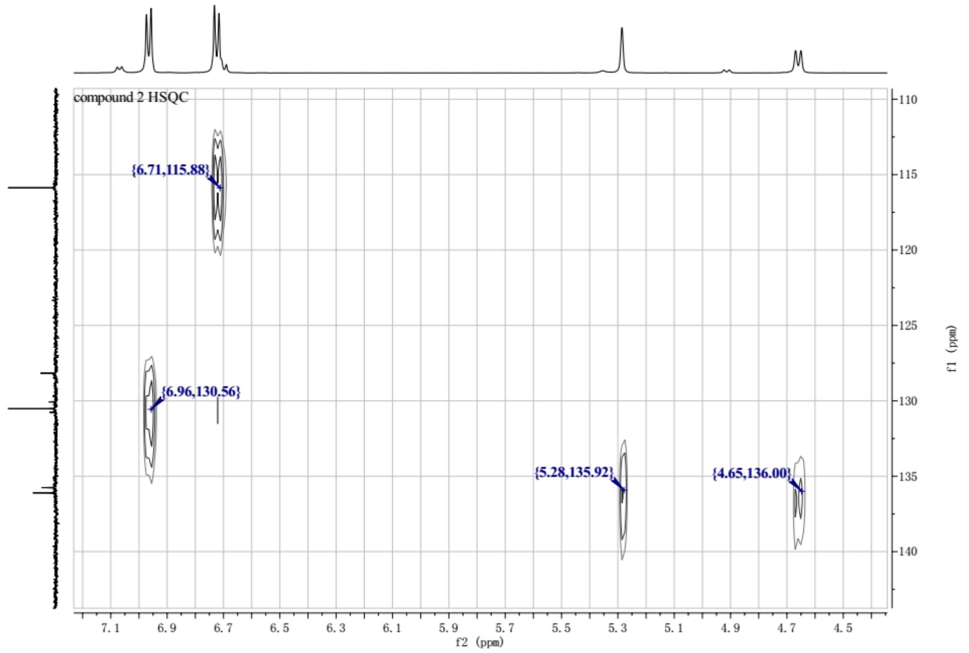

Figure S28. Slice 2 of the HSQC (500 MHz, $CDCl_3$-*d*) spectrum of compound **2**

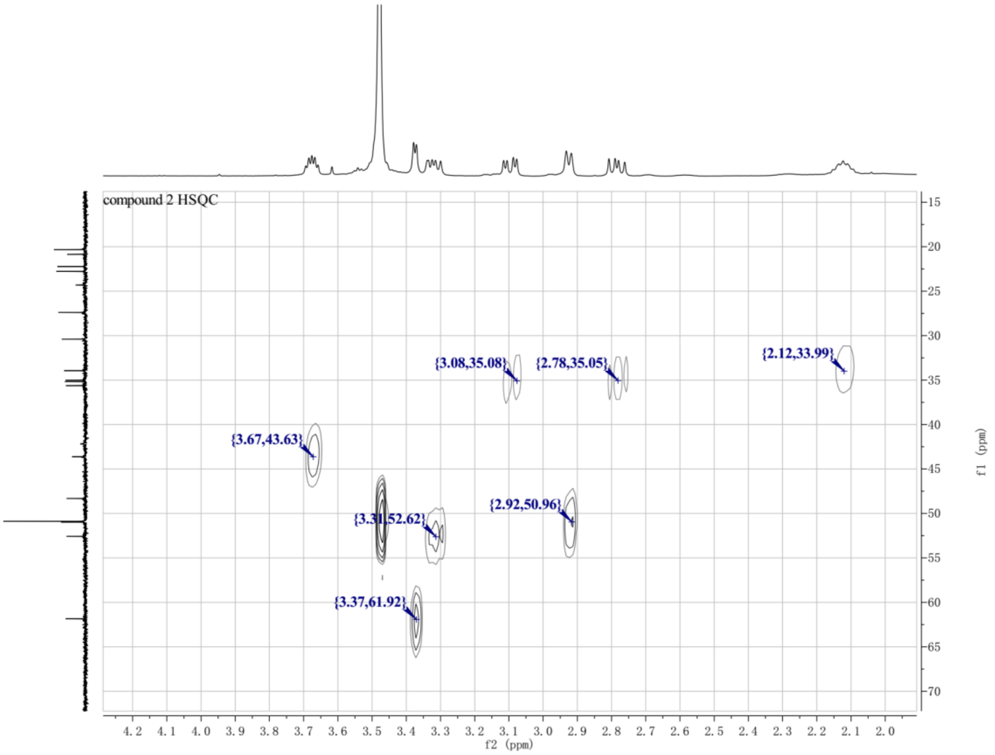

Figure S29. Slice 3 of the HSQC (500 MHz, $CDCl_3$-*d*) spectrum of compound **2**

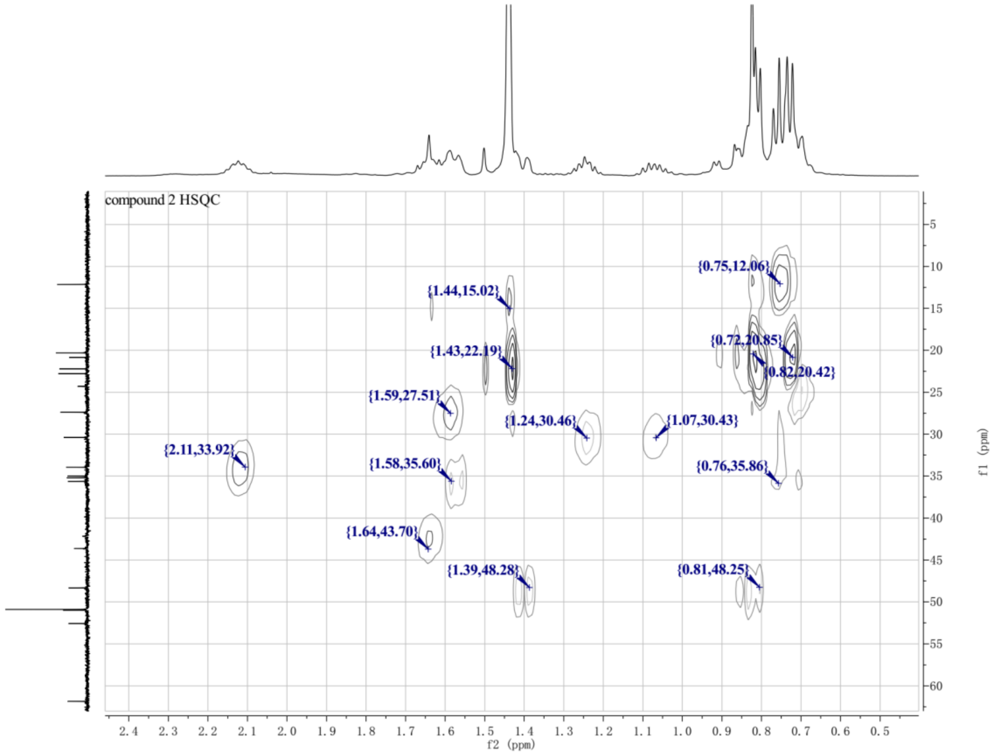

Figure S30. The HMBC (500 MHz, $CDCl_3$-*d*) spectrum of compound **2**

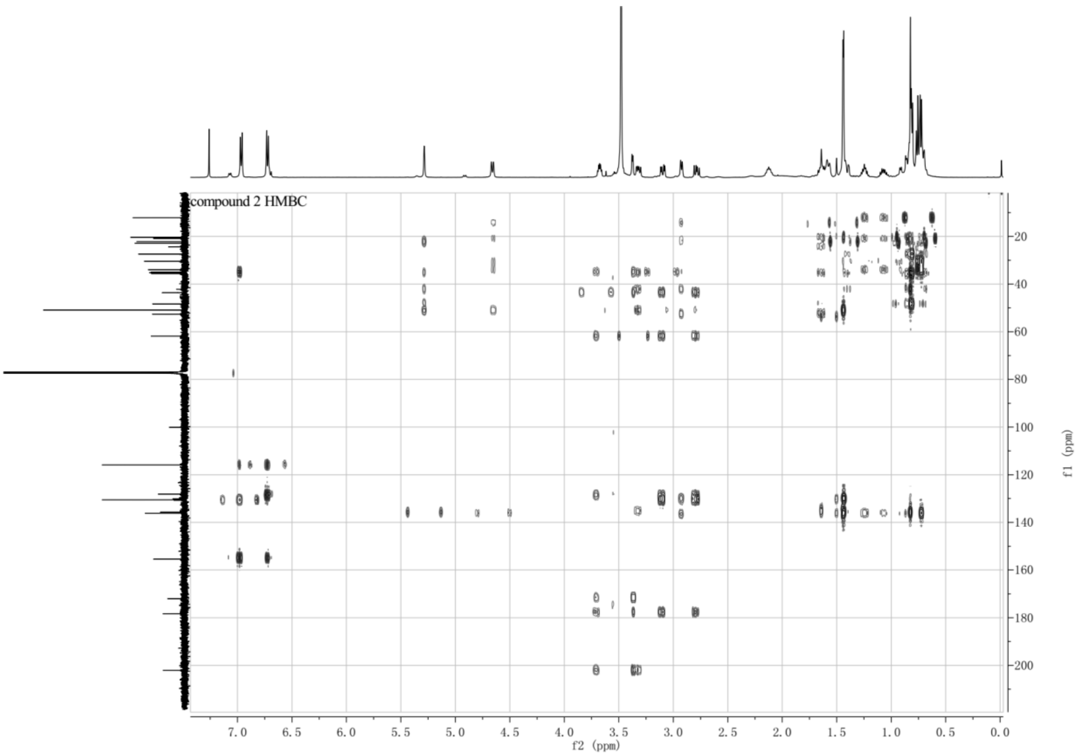

Figure S31. Slice 1 of the HMBC (500 MHz, $CDCl_3$-*d*) spectrum of compound **2**

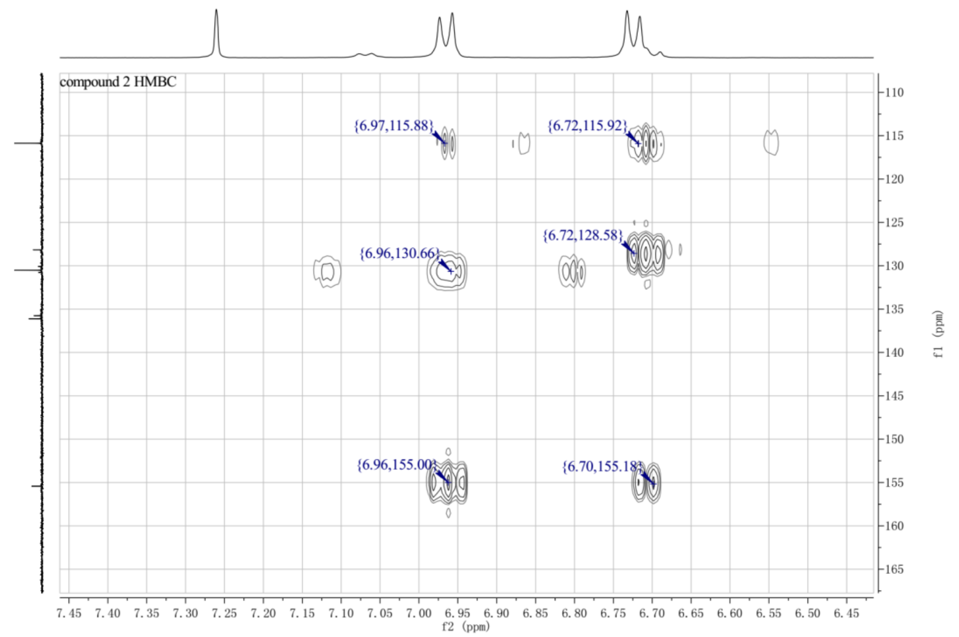

Figure S32. Slice 2 of the HMBC (500 MHz, $CDCl_3$-*d*) spectrum of compound **2**

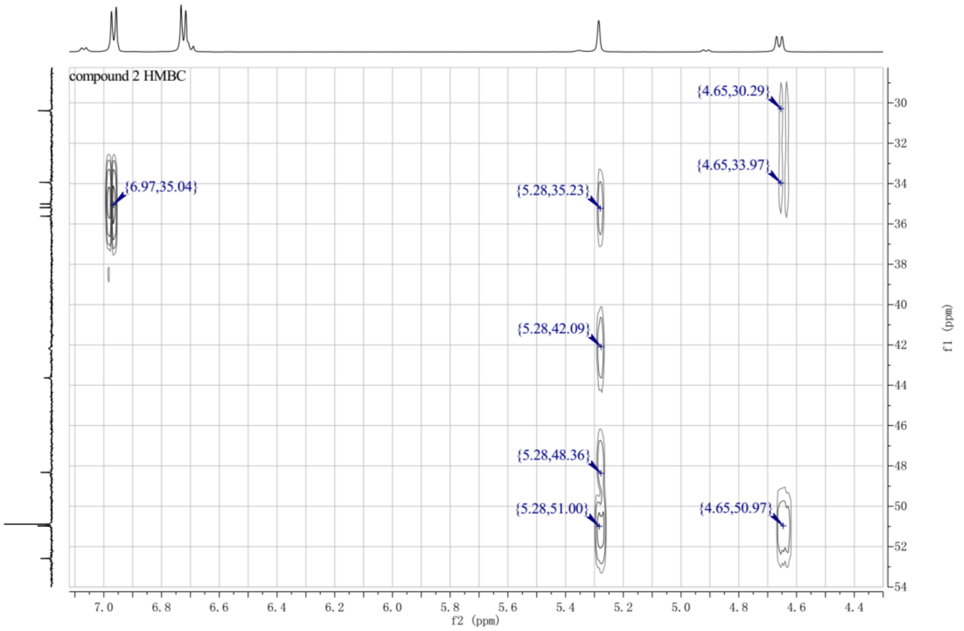

Figure S33. Slice 3 of the HMBC (500 MHz, $CDCl_3$-*d*) spectrum of compound **2**

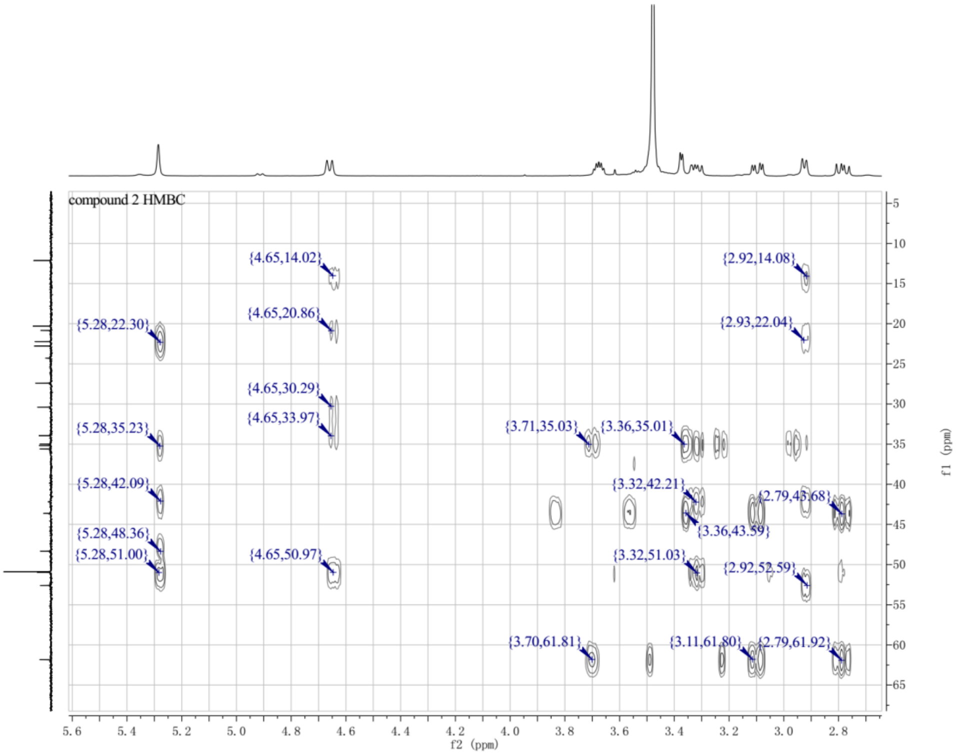

Figure S34. Slice 4 of the HMBC (500 MHz, $CDCl_3$-*d*) spectrum of compound **2**

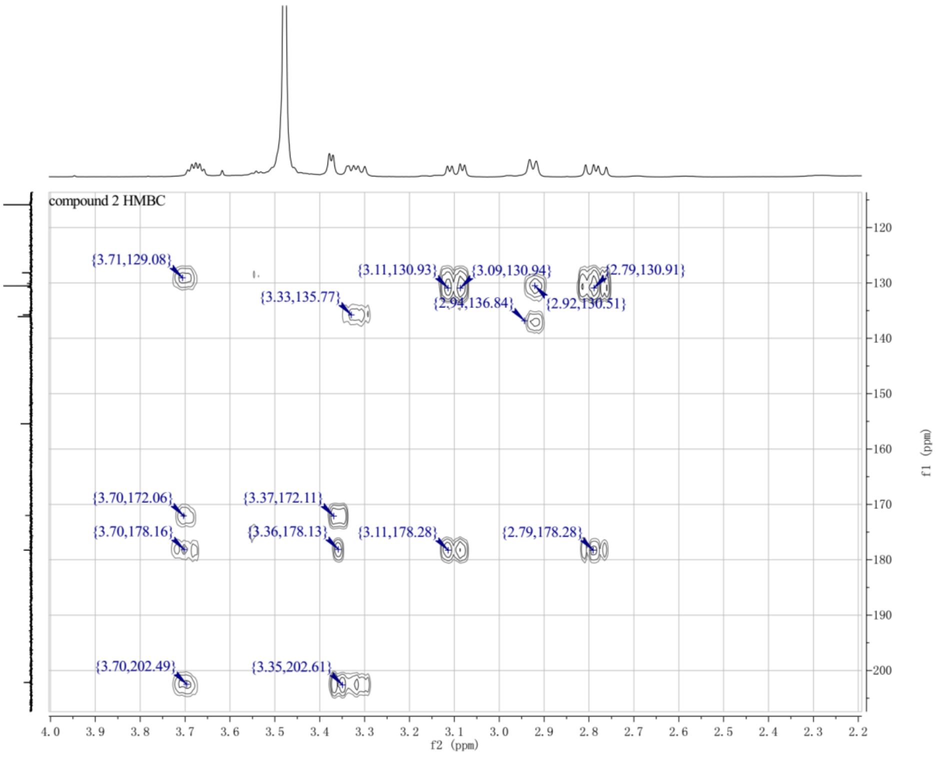

Figure S35. Slice 5 of the HMBC (500 MHz, $CDCl_3$-*d*) spectrum of compound **2**

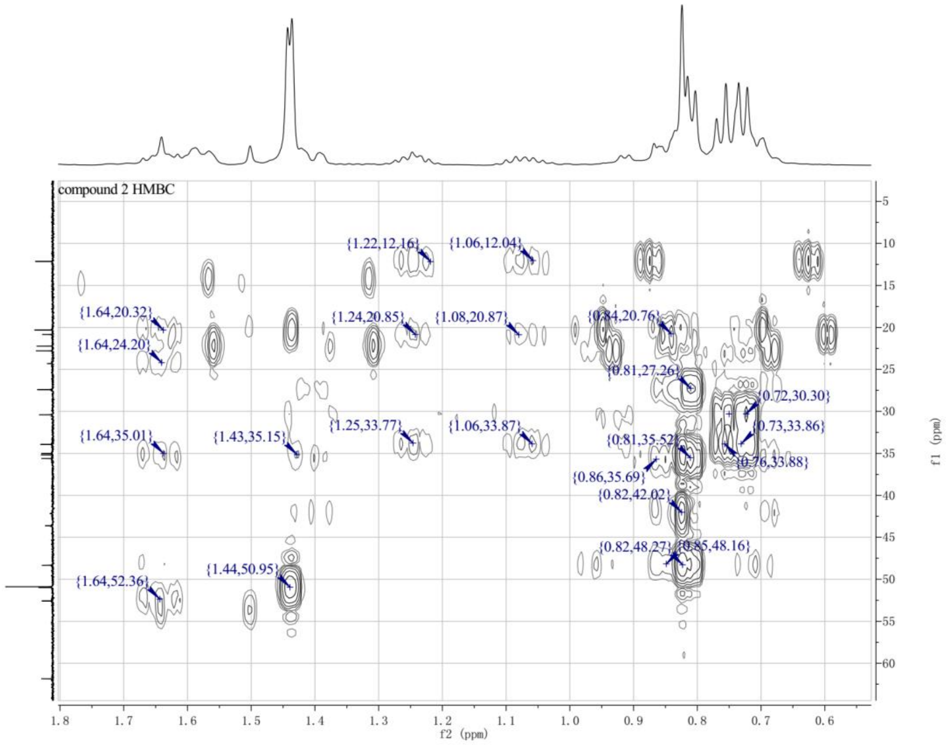

Figure S36. Slice 6 of the HMBC (500 MHz, $CDCl_3$-*d*) spectrum of compound **2**

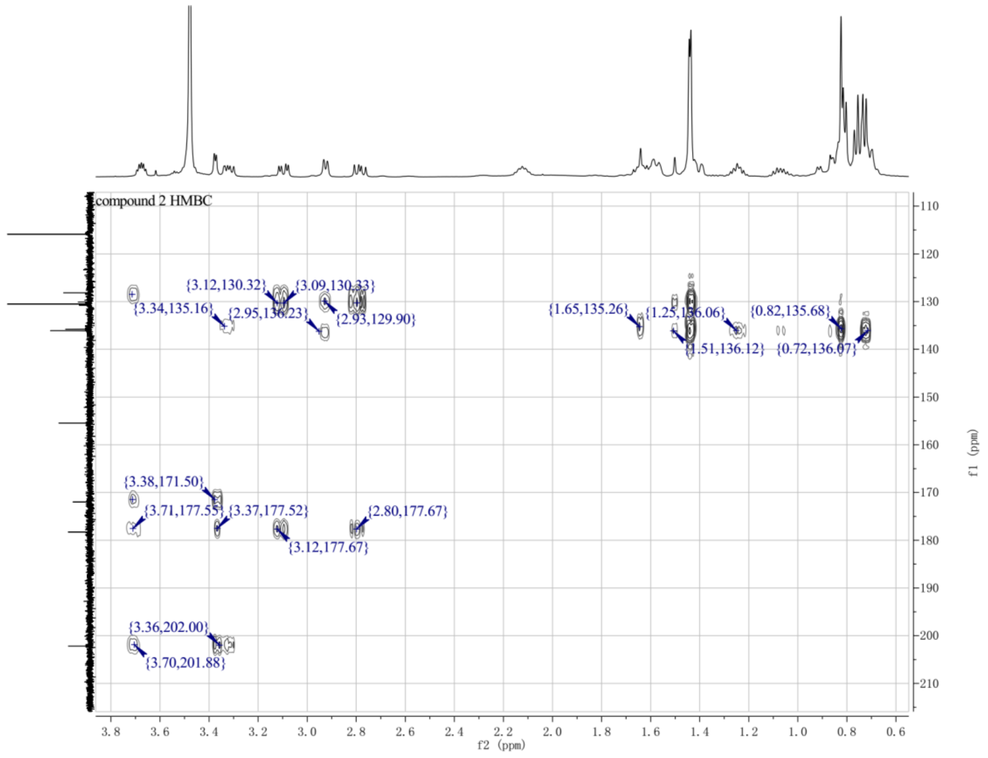

Figure S37. The ROESY (500 MHz, $CDCl_3$-*d*) spectrum of compound **2**

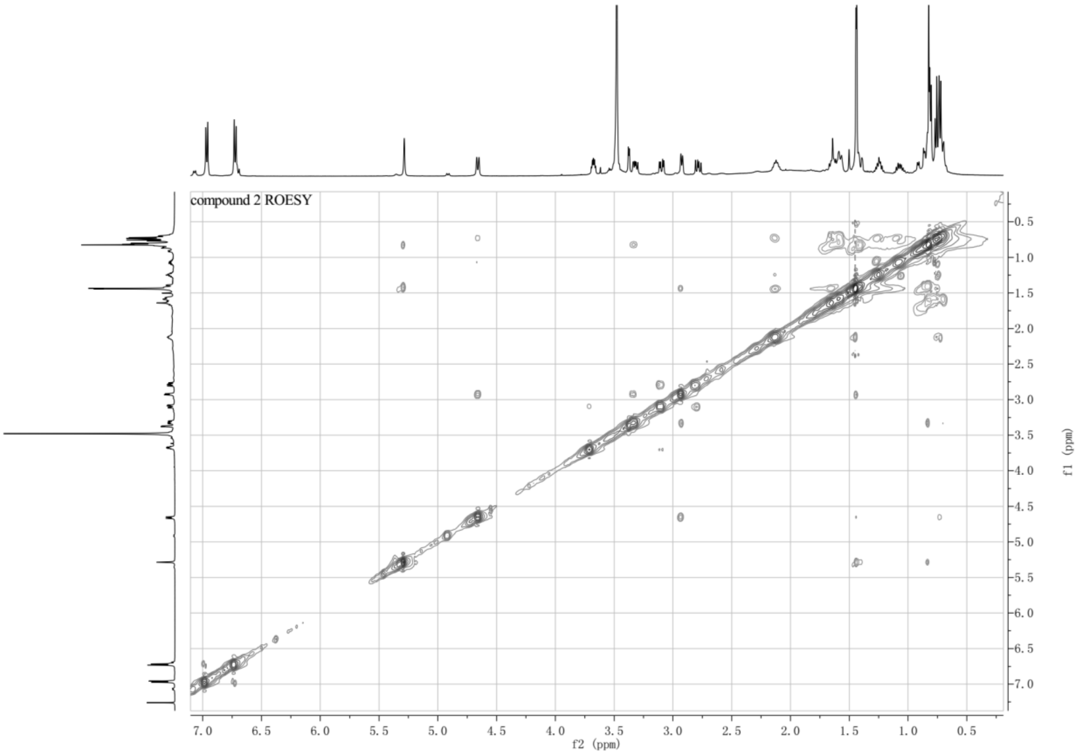

Figure S38. Slice 1 of the ROESY (500 MHz, $CDCl_3$-*d*) spectrum of compound **2**

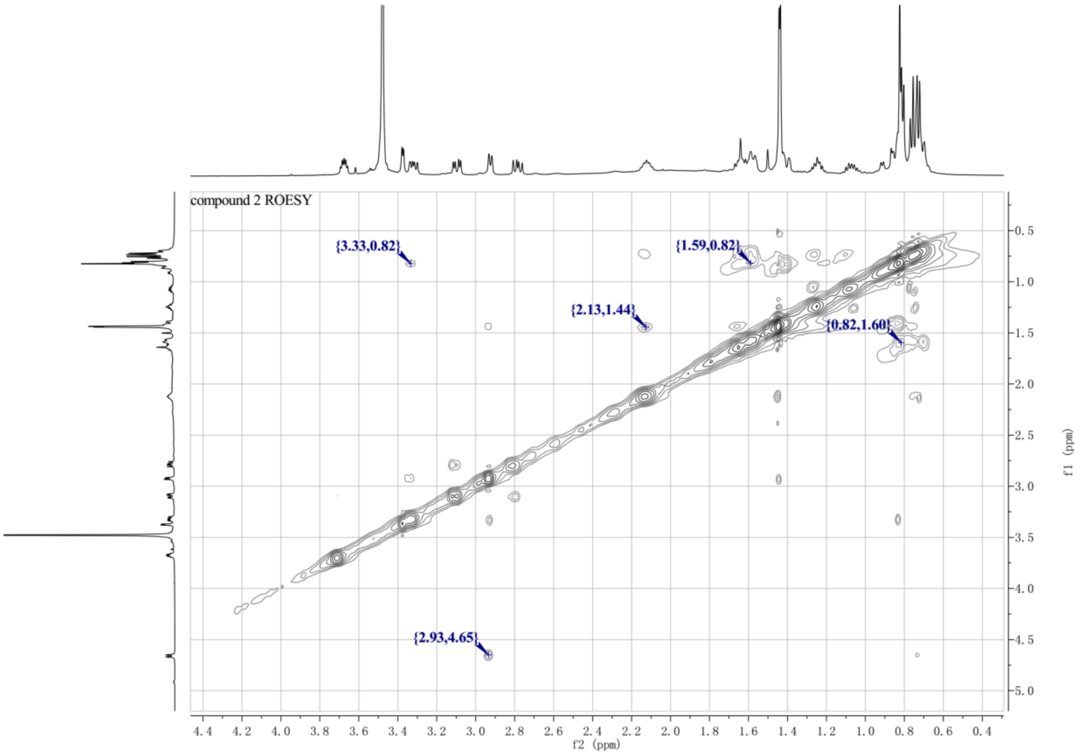

Figure S39. The HRESIMS spectrum of compound **2**

Qualitative Analysis Report

**Data Filename**	MG-13.d	**Sample Name**	MG-13
**Sample Type**	Sample	**Position**	P1-A8
**Instrument Name**	Instrument 1	**User Name**	
**Acq Method**	SIBU.m	**Acquired Time**	3/30/2017 2:50:39 PM
**IRM Calibration Status**	Success	**DA Method**	Default.m
**Comment**			

Sample Group **Info.**
Acquisition SW Version 6200 series TOF/6500 series Q-TOF B.05.01 (B5125.2)

User Spectra

Fragmentor Voltage	Collision Energy	Ionization Mode
175	0	ESI

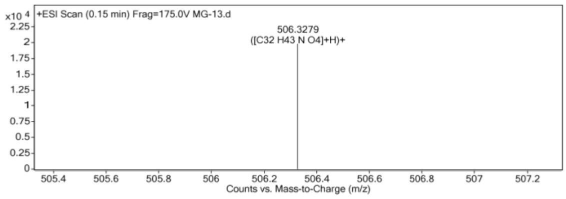

Peak List

m/z	z	Abund	Formula	Ion
81.9373		11158.62		
98.9865		15399.51		
107.0492	1	7656.9		
121.0509		8018.43		
214.9181		9841.88		
506.3279	1	19716.21	C32 H43 N O4	(M+H)+
528.3101	1	18128.14		
566.3981	1	8953.85		

Formula Calculator Element Limits

Element	Min	Max
C	3	60
H	0	120
O	0	30
N	0	3

Formula Calculator Results

Formula	CalculatedMass	CalculatedMz	Mz	Diff. (mDa)	Diff. (ppm)	DBE
C32 H43 N O4	505.3192	506.3265	506.3279	-1.6	-3.2	12.0000

--- End Of Report ---

Agilent Technologies Page 1 of 1 Printed at: 2:53 PM on: 3/30/2017

Figure S40. The ^{1}H NMR (500 MHz, CD_3OH-d_4) spectrum of compound **3**

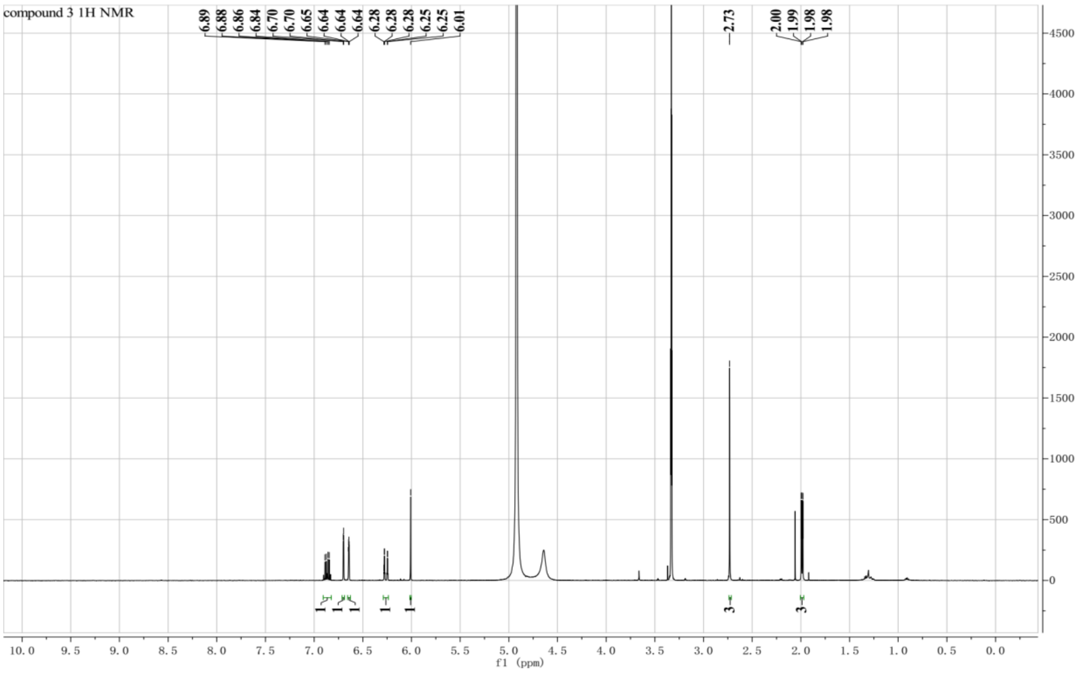

Figure S41. Enlarged ^{1}H NMR (500 MHz, CD_3OH-d_4) spectrum of compound **3**

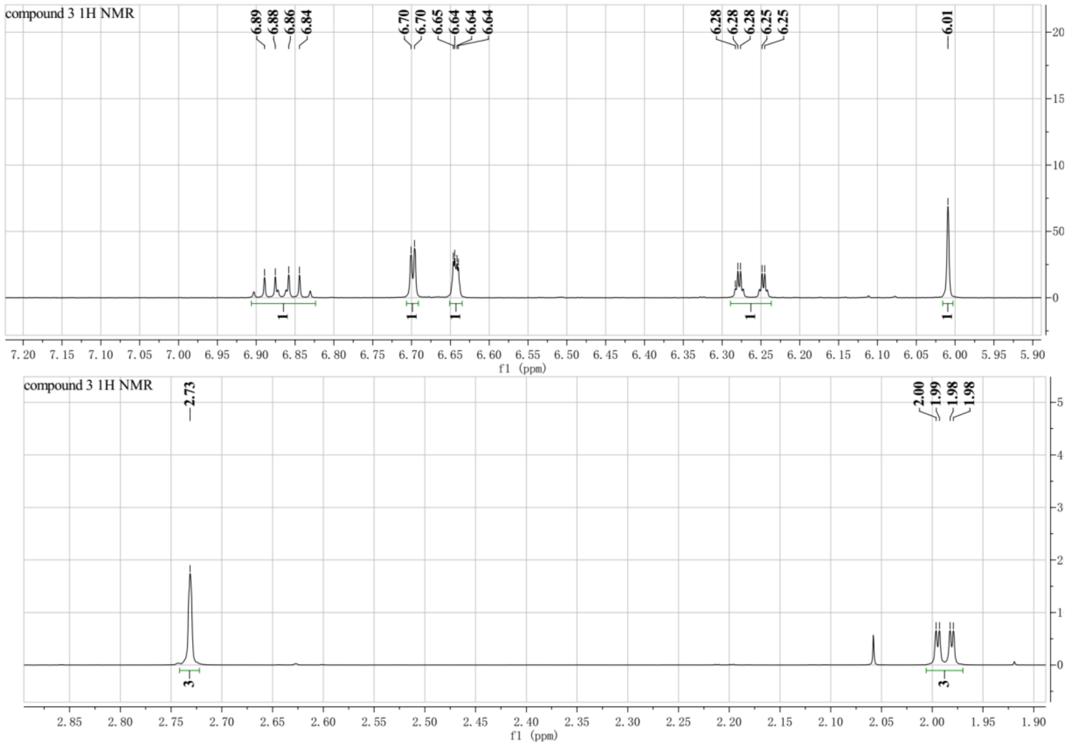

Figure S42. The ^{13}C NMR and DEPT-135 (125 MHz, CD_3OH-d_4) spectrum of compound **3**

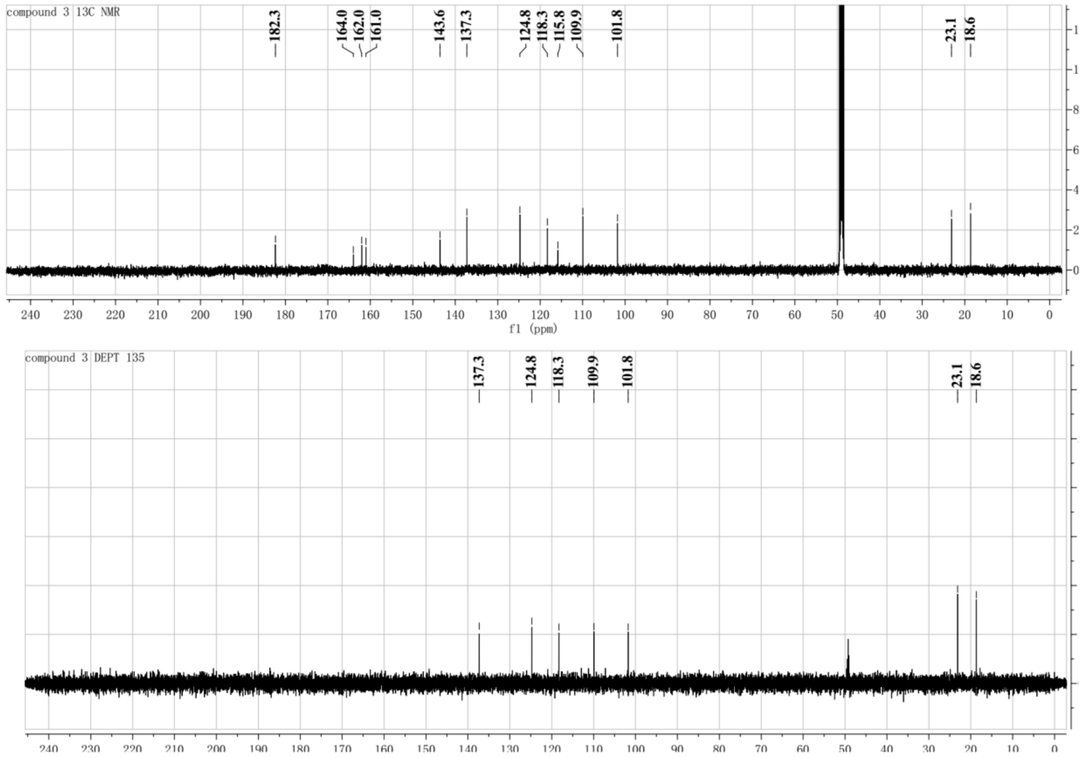

Figure S43. The ^{1}H-^{1}H COSY (500 MHz, CD_3OH-d_4) spectrum of compound **3**

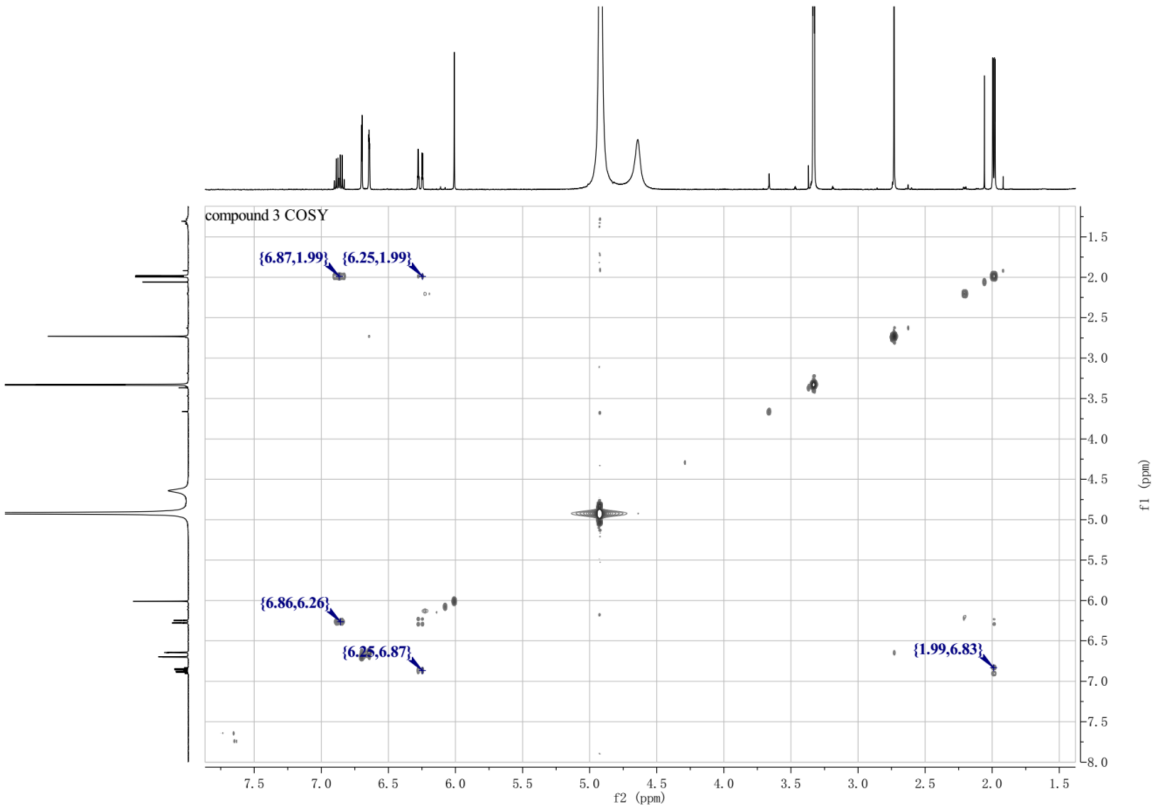

Figure S44. The HSQC (500 MHz, CD_3OH-d_4) spectrum of compound **3**

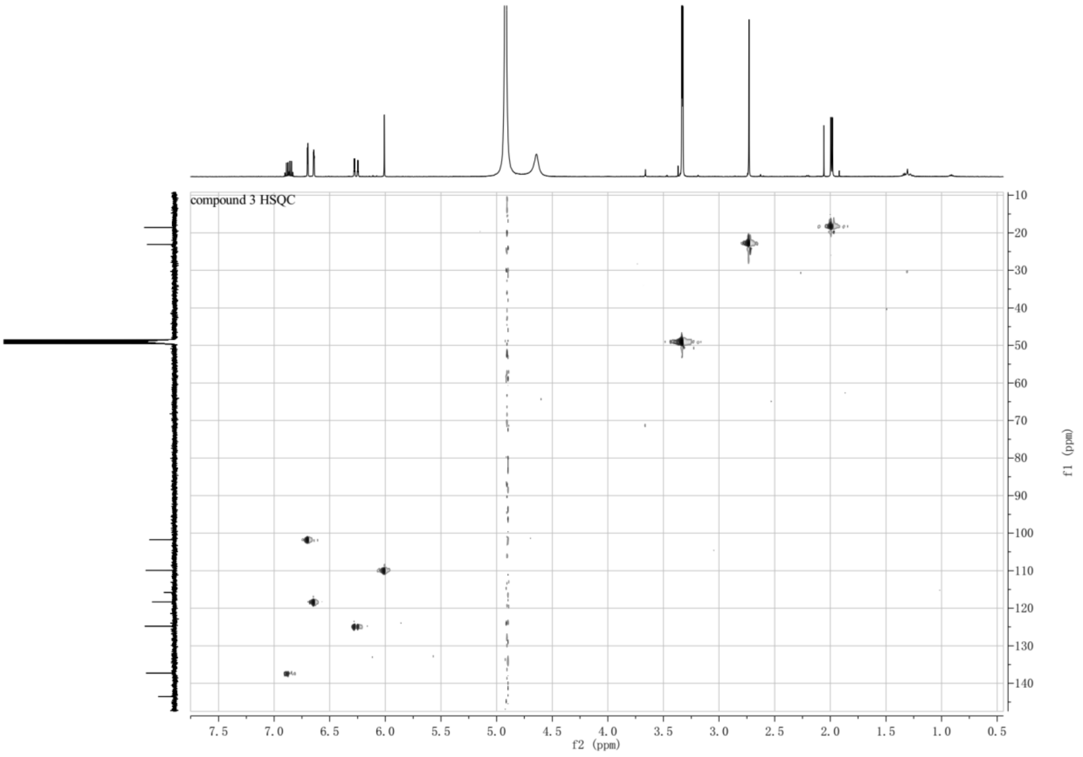

Figure S45. Slice 1 of HSQC (500 MHz, CD_3OH-d_4) spectrum of compound **3**

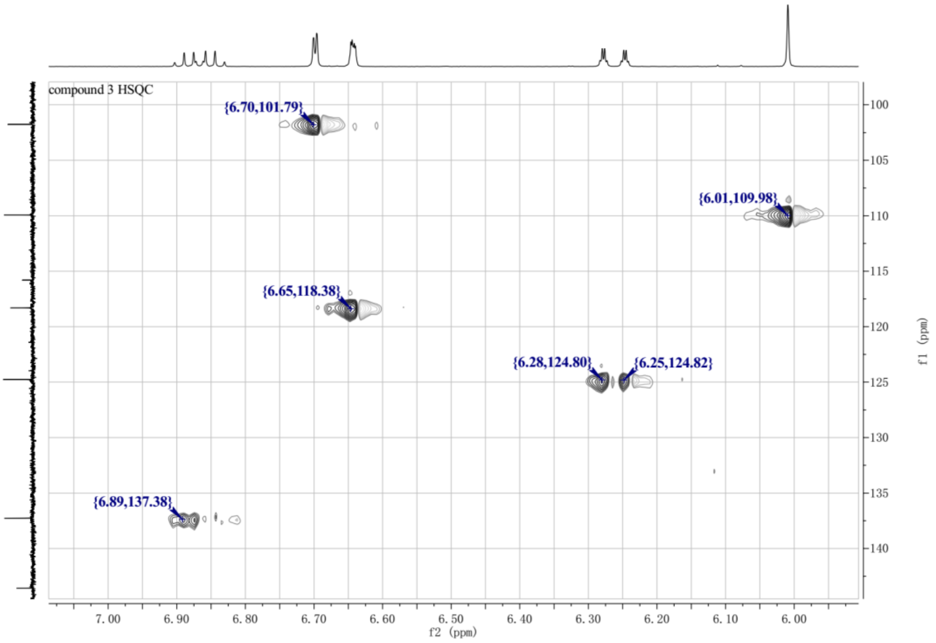

Figure S46. Slice 2 of HSQC (500 MHz, CD_3OH-d_4) spectrum of compound **3**

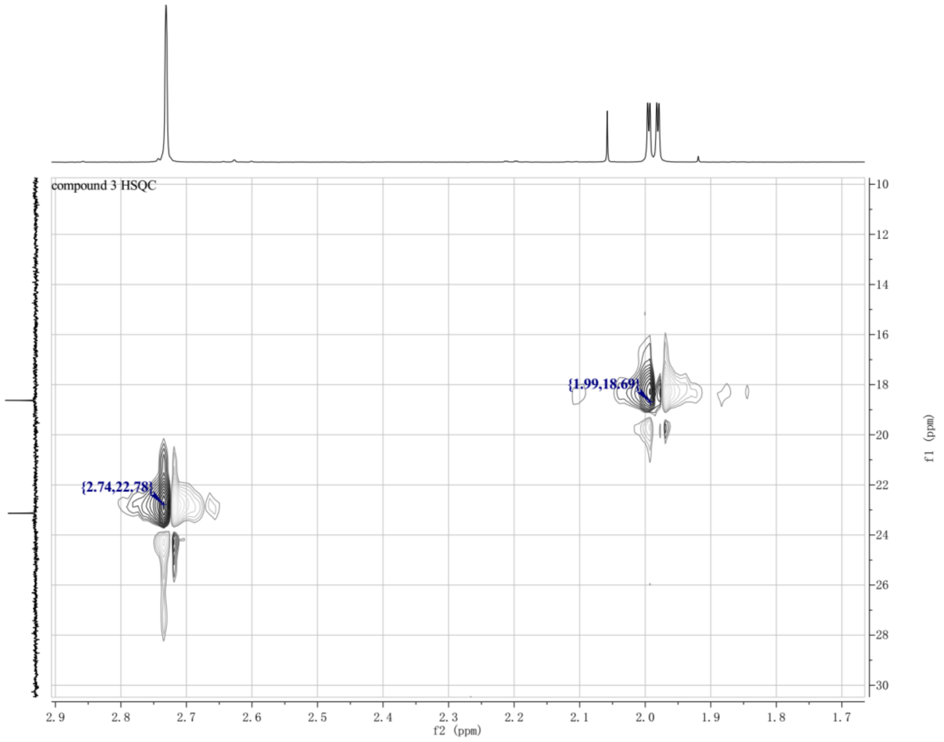

Figure S47. The HMBC (500 MHz, CD_3OH-d_4) spectrum of compound **3**

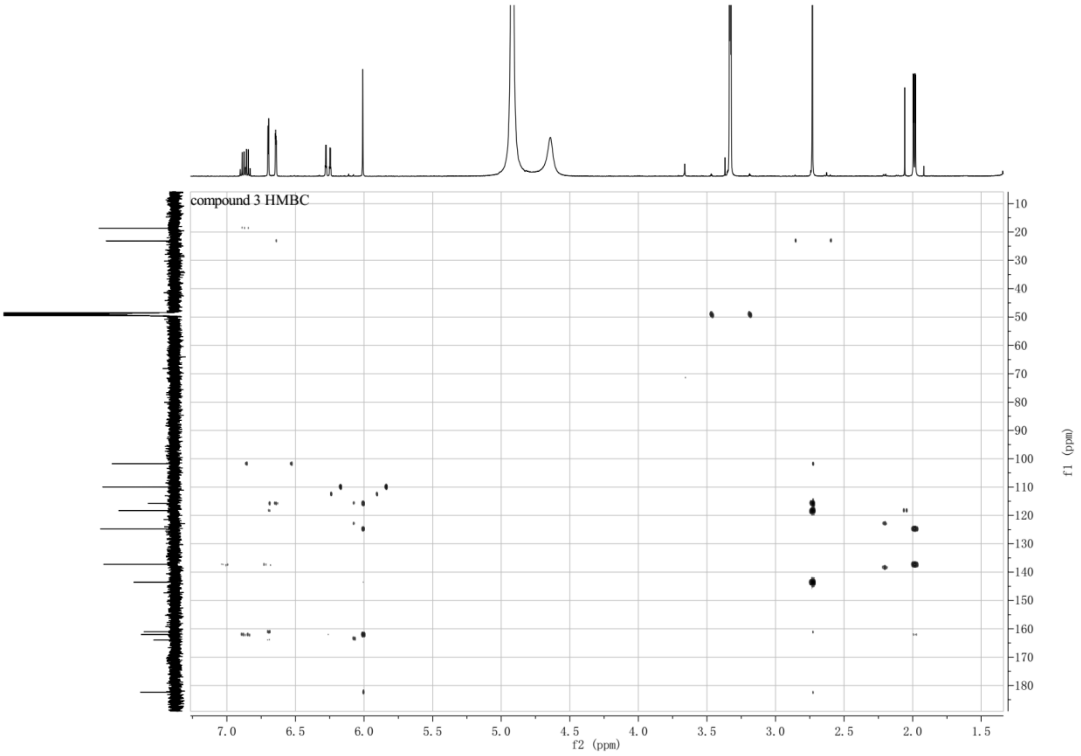

Figure S48. Slice 1 of HMBC (500 MHz, CD_3OH-d_4) spectrum of compound **3**

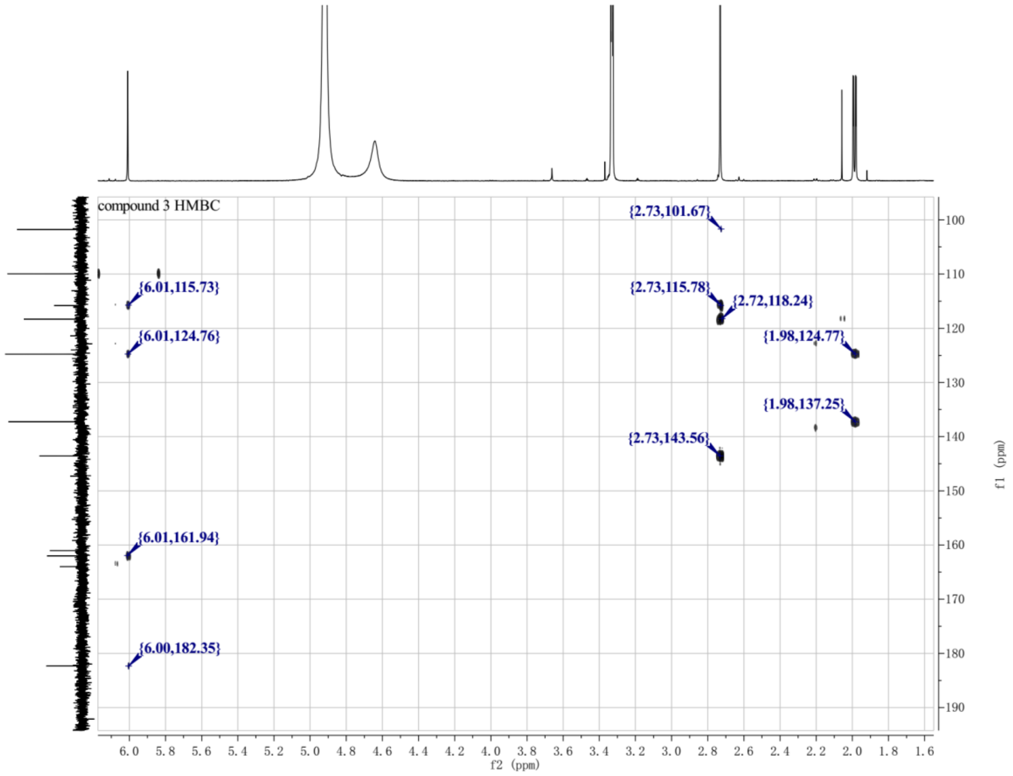

Figure S49. Slice 2 of HMBC (500 MHz, CD_3OH-d_4) spectrum of compound **3**

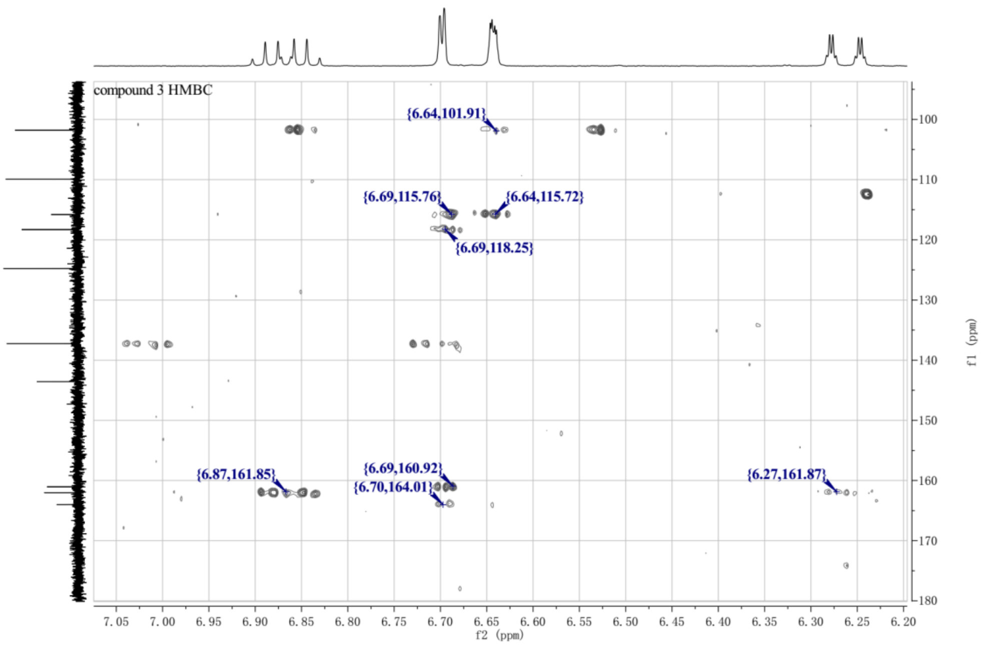

Figure S50. The HRESIMS spectrum of compound **3**

Qualitative Analysis Report

**Data Filename**	HK-DHF-7.d	**Sample Name**	HK-DHF-7
**Sample Type**	Sample	**Position**	P1-B3
**Instrument Name**	Instrument 1	**User Name**	
**Acq Method**	s-HRp.m	**Acquired Time**	3/13/2017 2:59:56 PM
**IRM Calibration Status**	Success	**DA Method**	sibu.m
**Comment**			

Sample Group **Info.**
Acquisition SW 6200 series TOF/6500 series
Version Q-TOF B.05.01 (B5125.2)

User Spectra

Fragmentor Voltage	Collision Energy	Ionization Mode
135	0	ESI

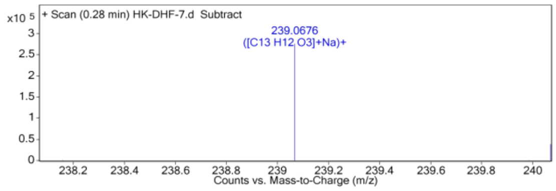

Peak List

m/z	z	Abund	Formula	Ion
163.123	1	29683.05		
217.086	1	163146.16		
218.09	1	24185.5		
239.068	1	275535.59	C13 H12 O3	(M+Na)+
240.071	1	38222.15	C13 H12 O3	(M+Na)+
255.041	1	30965.79		
455.147	1	110273.73		
456.15	1	29068.76		

Formula Calculator Element Limits

Element	Min	Max
C	3	60
H	0	120
O	0	30

Formula Calculator Results

Formula	CalculatedMass	CalculatedMz	Mz	Diff. (mDa)	Diff. (ppm)	DBE
C13 H12 O3	216.0786	239.0679	239.0676	0.2	1.0	8.0000

--- End Of Report ---
